# Thiol-free chemoenzymatic synthesis of β-ketosulfides

**DOI:** 10.3762/bjoc.15.34

**Published:** 2019-02-11

**Authors:** Adrián A Heredia, Martín G López-Vidal, Marcela Kurina-Sanz, Fabricio R Bisogno, Alicia B Peñéñory

**Affiliations:** 1 INFIQC-CONICET, Departamento de Química Orgánica, Facultad de Ciencias Químicas, Universidad Nacional de Córdoba. Ciudad Universitaria, Córdoba, X5000HUA, Argentina; 2 INTEQUI-CONICET, Área de Química Orgánica, Facultad de Química, Bioquímica y Farmacia, UNSL. Chacabuco y Pedernera, San Luis, 5700, Argentina

**Keywords:** ketosulfides, lipase, multicomponent, sulfoxide, thiol-free

## Abstract

A preparation of β-ketosulfides avoiding the use of thiols is described. The combination of a multicomponent reaction and a lipase-catalysed hydrolysis has been developed in order to obtain high chemical diversity employing a single sulfur donor. This methodology for the selective synthesis of a set of β-ketosulfides is performed under mild conditions and can be set up in one-pot two-step and on a gram-scale.

## Introduction

Throughout the years, several strategies have been developed to build up organic compounds bearing a sulfide moiety [1–2]. Often, thiols (or the corresponding thiolate anions) are employed as nucleophilic sulfur reagents in order to react with a suitable electrophile [3–4], however, there are certain negative aspects of thiols that need to be taken into account (i.e., foul smell, easy oxidation into disulfide, participation as donors in one-electron events, reaction with olefins through ene-type reactions, etc) [5–8]. Hence, the development of thiol-free protocols for the synthesis of organosulfur compounds is highly desirable [9].

**Figure 1:**
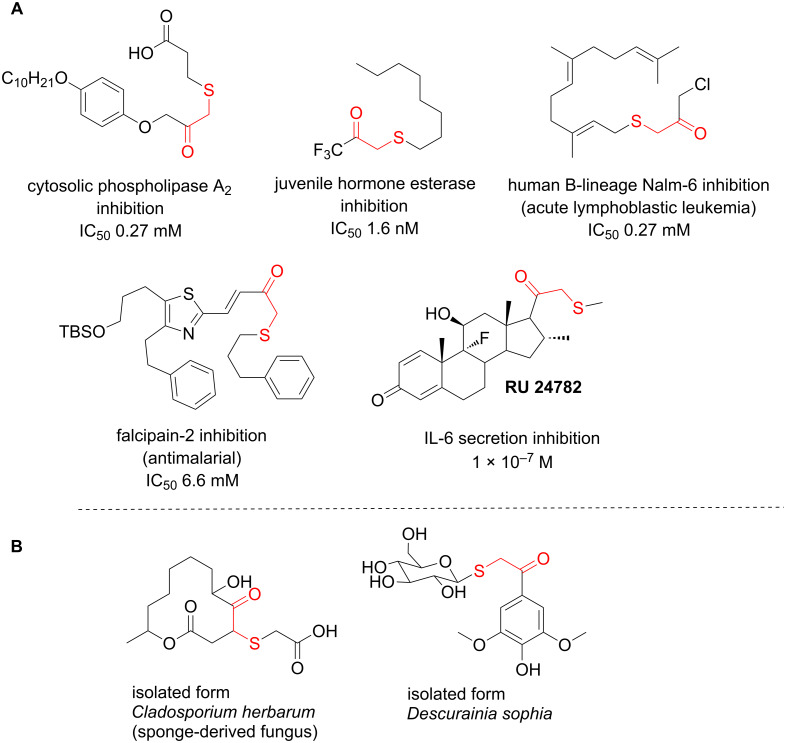
Selected examples of valuable β-ketosulfides. A: bioactive synthetic compounds, B: natural products.

In particular, the β-ketosulfide motif is present in natural products [10–11] and synthetic compounds displaying important bioactivities (Figure 1) [12–16].

**Scheme 1:**
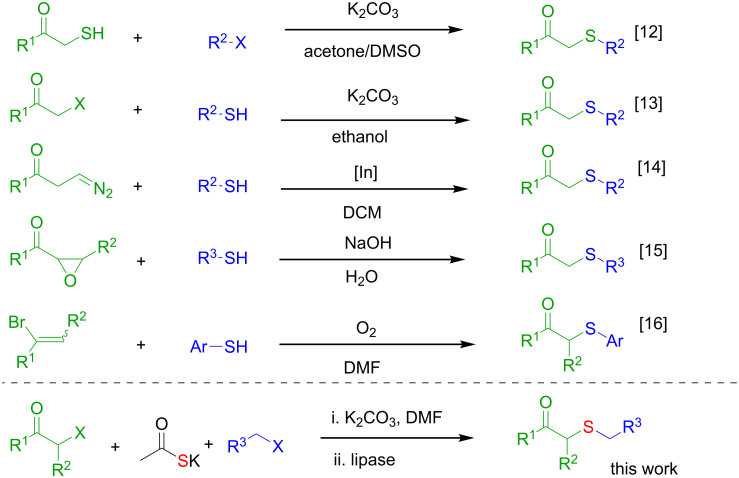
Different strategies for the preparation of β-ketosulfides.

Besides the well-established protocols that make use of thiols (Scheme 1) [17–21], other methodologies employing different sulfur sources such as disulfides or silylsulfides have been described, which most of them involves metals, e.g., indium [22], copper [23], mercury [24], or organocatalysts [25].

β-Ketosulfides, in addition, play an important role as precursors in the synthesis of bioactive compounds [13,26–27], substrates for multicomponent reactions [28], and lately, have successfully been applied in polymer photodecoration [29–30]. They can easily be reduced into chiral hydroxy derivatives [31] and properly oxidised at sulfur to generate the corresponding chiral sulfoxides or sulfone derivatives [32].

Multicomponent reactions (MCRs), in which three or more reagents react giving rise to generally complex molecules in one-pot, have arisen as a powerful tool to connect fragments in a simple manner, avoiding cost and time-consuming isolation of intermediates and creating chemical diversity with high atom economy [33–36].

In the last two decades, enzymes have found a privileged place in organic chemistry by virtue of its inherent selectivity and eco-friendly reaction conditions. Such properties, among other, make them catalysts of choice for multiple kilo- and even ton-scale industrial processes [37–38].

The combination of MCRs and enzymatic catalysis offers a myriad of new possibilities by taking advantage of the robustness and bond forming power of MCRs and the mildness and selectivity displayed by biocatalysts [39–40]. Such a combination has been scarcely exploited as compared to strategies comprising transition metal catalysis and biocatalysis [41] or, in a lesser extent, organocatalysis and enzymes [42].

**Scheme 2:**
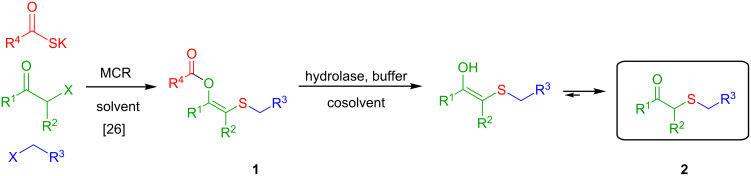
Thiol-free chemoenzymatic synthesis of β-ketosulfides.

In this context, a versatile and robust synthesis of β-ketosulfides avoiding the use of thiols under benign conditions is highly desirable. Based on our previously developed MCR [43] for the synthesis of enol esters with sulfur-containing substituents, we envisaged a two-step methodology starting from an α-haloketone, a thiocarboxylate and an alkyl (pseudo)halide (Scheme 2). Thus, once the enolester is formed, an enzyme-catalysed hydrolysis and protonation of the resulting enolate would render the title β-ketosulfide products. This strategy avoids the use of acidic or basic conditions for the hydrolysis of the ester moiety that, normally, result unsuitable for methylene active-containing products as β-ketosulfides [44].

## Results and Discussion

In a first set of experiments, a series of commercially available hydrolases were screened in aqueous buffer containing 5% v/v of an organic cosolvent towards a β-thioalkyl-substituted enolester as model substrate **1a** (Table 1). As enol acetates, (e.g., vinyl acetate, isopropenyl acetate), are outstanding acyl donors in lipase-catalysed reactions [45], it is expected that the desired lipase-catalysed hydrolysis shall be controlled by the steric demand of substituents at the enoyl moiety. On the other hand, cosolvent and buffer composition are two factors that may influence both, substrate solubility and enzyme activity. Hence, two different buffer solutions (KPi 50 mM pH 7.5 and Tris·HCl 50 mM pH 7.5) containing 5% v/v of an (miscible or inmiscible) organic cosolvent, were tested towards a set of commercially available lipases. In this line, a remarkable hydrolytic activity (89–99% conversion) was found for *Candida antarctica* lipase B (CAL-B, Novozym^®^ 435) in all tested conditions (Table 1, entry 2). For porcine pancreas lipase (PPL), a different scenario was found, since conversion was strongly influenced by buffer composition and, to a lesser extent, the cosolvent nature. For instance, when toluene was tested as cosolvent, conversions varied from 40% (in KPi) to 9% in (Tris·HCl), and for DMSO conversions range from 90% (in KPi) to 20% (Tris·HCl, Table 1, entry 4). *Candida rugosa* lipase (CRL, entry 5) and immobilised *Burkholderia cepacia* lipase (PSL-IM, Table 1, entry 3) displayed lower activities (3–26% and 6–32% conversion, respectively). Meanwhile, *Candida antarctica* lipase A (CAL-A, Table 1, entry 1), immobilised *Thermomyces lanuginosa* (Lypozyme TL IM, Table 1, entry 6) and *Rhizomucor miehei* lipase (Lypozyme RM, Table 1, entry 7) showed marginal or no activity in the tested conditions.

**Table 1:** Screening of hydrolases and conditions towards a model substrate.^a^

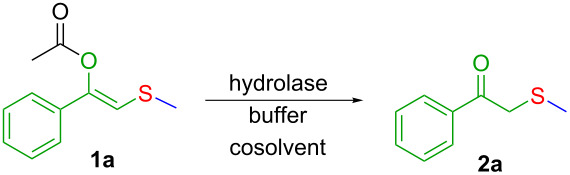									

entry	hydrolase	MeCN 5%		DMSO 5%		toluene 5%		MTBE 5%	
		Tris·HCl	KPi	Tris·HCl	KPi	Tris·HCl	KPi	Tris·HCl	KPi

1	CAL-A	<1	<1	<1	3	1	1	2	1
2	CAL-B	**93**	**89**	**90 (99)**^b^	**>99**	**97**	**97**	**>99**	**93**
3	PSL-IM	7	14	6	32	16	16	10	13
4	PPL	24	26	20	**90**	9	41	25	29
5	CRL	26	3	3	19	7	7	20	26
6	Lypozyme TL-IM	1	<1	<1	2	2	2	5	4
7	Lypozyme RM	2	2	<1	5	2	3	2	2
8	–	<1	<1	<1	<1	<1	<1	<1	<1

^a^Reaction conditions: 3 mg of **1a** (30 mM) and 3 mg of enzyme, cosolvent 5% v/v, final volume 500 μL, 12 h; conversion was determined by GC-FID % relative area; ^b^1.25 mg of **1a** (final conc. 12 mM) and 2 mg of enzyme.

In general, no clear correlation could be drawn by considering the cosolvent logP (or water miscibility) and buffer nature with the lipase activity towards the model substrate **1a**.

**Figure 2:**
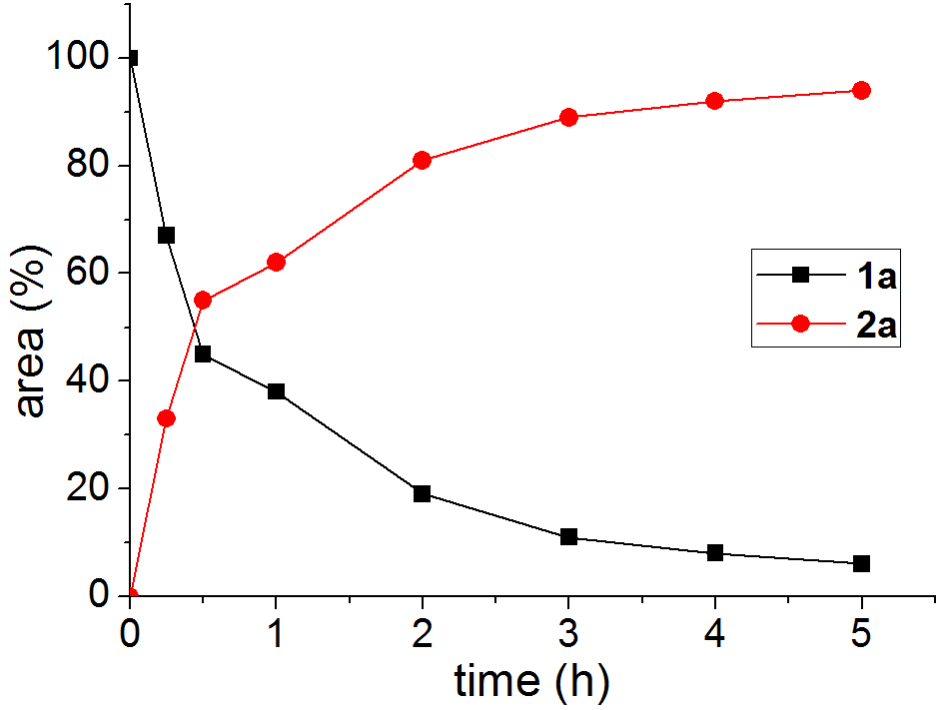
Time-course plot for the CAL-B catalysed hydrolysis of **1a**.

Once the best conditions were set, the model reaction was monitored as a function of time (Figure 2). As can be seen, the biohydrolysis of **1a** reaches around 80% conversion at 2 h and is complete after 8 h.

Next, a series of diversely substituted β-thioalkyl enol esters was submitted to CAL-B-catalysed hydrolysis under the chosen conditions, as summarised in Table 2.

**Table 2:** Lipase-catalysed hydrolysis–protonation sequence over a series of β-thioalkyl enol esters.^a^

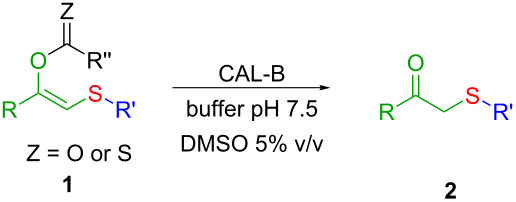			

entry	substrate	product	conv. %^b^

1	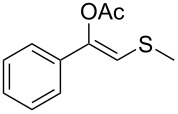 **1a**	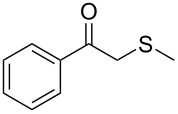 **2a**	>99
2	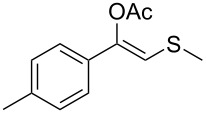 **1b**	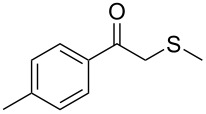 **2b**	>99
3	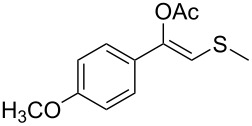 **1c**	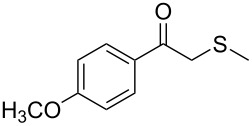 **2c**	>99
4	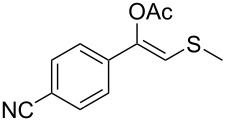 **1d**	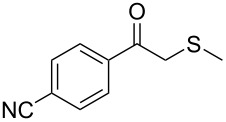 **2d**	94
5	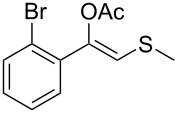 **1e**	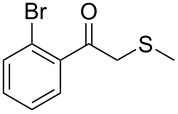 **2e**	>99
6	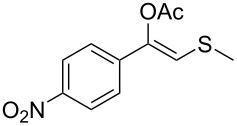 **1f**	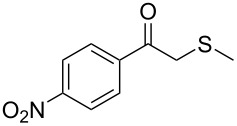 **2f**	40 (96)^c^
7	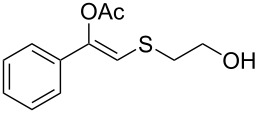 **1g**	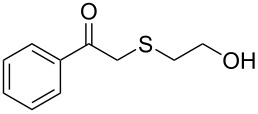 **2g**	>99
8	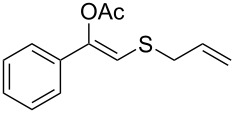 **1h**	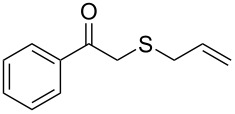 **2h**	98
9	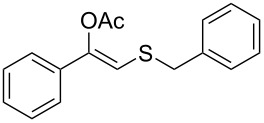 **1i**	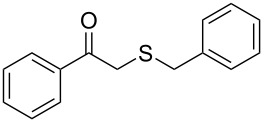 **2i**	41 (94)^d^
10	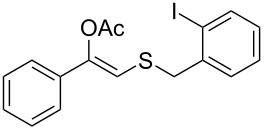 **1j**	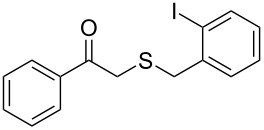 **2j**	19
11	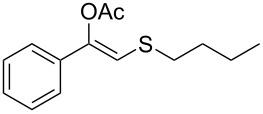 **1k**	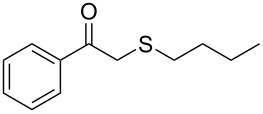 **2k**	62
12	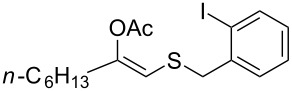 **1l**	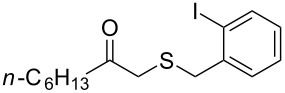 **2l**	31 (61)^c^
13	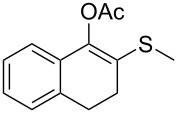 **1m**	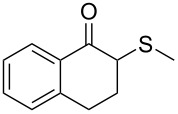 **2m**	52 (95)^c^ (65)^e^
14	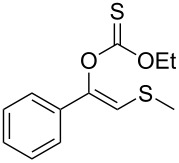 **1n**	–	n.r.
15	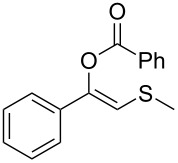 **1o**	–	n.r.
16	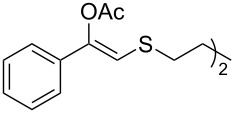 **1p**	–	n.r.
17	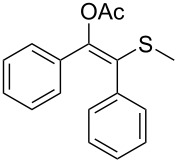 **1q**	–	n.r.
18	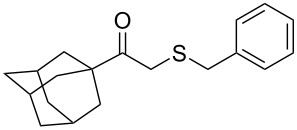 **1r**	–	n.r.
19	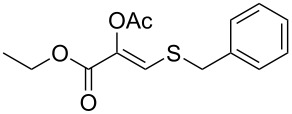 **1s**	–	n.r.
20	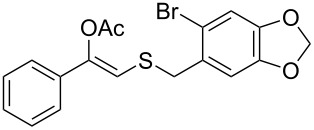 **1t**	–	n.r.
21	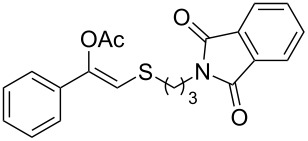 **1u**	–	n.r.
22	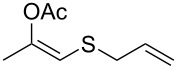 **1v**	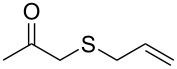 **2v**	>99
23	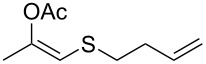 **1w**	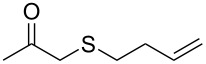 **2w**	>99
24	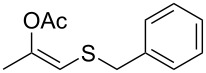 **1x**	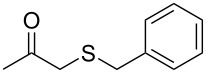 **2x**	>99

^a^Reaction conditions: Substrate **1a**–**x** (12 mM) and 2 mg of CAL-B (Novozym^®^ 435), final volume 500 μL, Tris·HCl buffer (50 mM pH 7.5), cosolvent DMSO 5% v/v, 30 °C, 24 h, 250 rpm; ^b^GC–FID % relative area; ^c^4 mg of enzyme; ^d^3 mg of enzyme; ^e^48 h reaction time; n.r.: no reaction.

As can be seen in Table 2, substrates containing diversely substituted aryl moieties at the α-position of the enol ester, underwent smooth conversion (typically 94–99% except for *p*-nitrophenyl derivative **1f**, for which more enzyme was needed to reach 96%, Table 2, entry 6), regardless the electronic nature of the substituents (see Table 2, entries 1–6) [46]. For aliphatic substituents at the α-position, a methyl group was perfectly accepted (Table 2, entries 22–24). Longer unbranched hydrocarbon was tolerated, although moderate conversion was achieved, even with higher lipase loading (Table 2, entry 12, **1l**). For the latter, it must be considered the additional bulky *o*-iodobenzyl substituent attached to the sulfur. The bulky alicyclic adamantyl α-substituent (compound **1r**), was not accepted as substrate (Table 2, entry 18). The same results were obtained when an ester moiety was pending at α-position (**1s**, Table 2, entry 19).

Substitution on the sulfur atom was also screened, and small substituents [such as methyl- (**1a**), 2-hydroxyethyl- (**1g**), allyl- (**1h**)] rendered excellent conversions (98–99%, Table 2, entries 1, 7, and 8, respectively). For a bulkier linear *S*-substituent (butyl), **1k**, the conversion dropped to 62% (Table 2, entry 11) and for *S*-Bn derivative **1i**, a slower reaction took place, reaching 41% conversion under standard conditions and an improved 94% conversion when the catalyst loading was risen to 50% (Table 2, entry 9). The *S*-(*o*-iodobenzyl) derivative **1j** (Table 2, entry 10) rendered a fair 19% conversion, considering the high steric demand of α- and ß-substituents. For even bulkier *S*-(bromobenzodioxole)methyl derivative **1t** (Table 2, entry 20) no conversion was detected at 24 h. Similar results were obtained with the *S*-propyl-3-phthalimido derivative **1u** (Table 2, entry 21) and the dimeric substrate bis-enol acetate **1p** (Table 2, entry 16), suggesting that *o*-iodobenzyl substituent is approaching the upper limit of steric congestion. Additional β-substitution could be tolerated in the tetralone-derived substrate, **1m**, affording 52 and 65% conversion at 24 and 48 h, respectively. Accordingly, 95% conversion at 24 h was achieved by a twofold increase of lipase loading (Table 2, entry 13). On the contrary, no conversion was detected for the α,β-diphenyl enol acetate substrate **1q** (Table 2, entry 17).

In order to test the chemoselectivity of the enzymatic hydrolysis, we turned our attention to the acyl moiety of the enol ester. Hence, only acetate was easily accepted while ethyl thiocarbonate **1n** (Table 2, entry 14) and benzoate enol esters **1o** (Table 2, entry 15), were recovered unaltered. These results pave the way for developments of orthogonal deprotection protocols in the future.

**Scheme 3:**
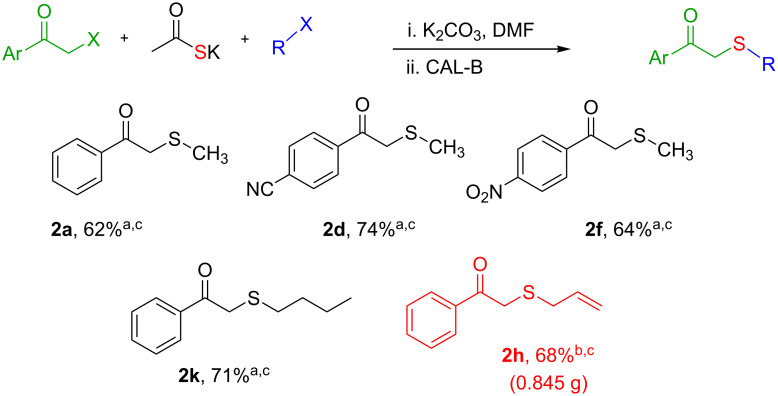
One-pot two-step preparation of phenacylalkylsulfides. ^a^Reaction conditions: i. α-haloketone (0.25 mmol), potassium thioacetate (1.1 equiv), alkyl halide (1.1 equiv) and K_2_CO_3_ (2.0 equiv) in 500 µL of DMSO, 5 h at room temperature; ii. 100 mg of CAL-B (Novozyme 435) and 9.5 mL of Tris·HCl buffer (50 mM pH 7.5), 30 °C, 24 h, 250 rpm. ^b^Reaction performed using 1.0 g of α-chloroacetophenone, potassium thioacetate (1.1 equiv), alkyl halide (1.1 equiv) and K_2_CO_3_ (2.0 equiv) in 5 mL of DMSO, 200 mg of CAL-B and 45 mL of Tris-HCl buffer (50 mM pH 7.5), 24 h at room temperature one-pot two-step preparation of the phenacyl allyl sulfide 2h. ^c^Isolated yield.

Once the enzymatic hydrolysis and the MCR reaction were optimised, we investigated the robustness of this protocol for the one-pot two-step preparation of different β-ketosulfide departing from the corresponding α-haloacetophenone at higher scale, as shown in Scheme 3, and isolated the products in good yields.

As depicted in Scheme 3, α-chloroacetophenone (1.0 g, 6.47 mmol) was reacted with 1.1 equiv of potassium thioacetate, 1.1 equiv of allyl bromide and 2 equiv of K_2_CO_3_ in 5 mL of DMSO. After 24 h, 45 mL of buffer (Tris·HCl 50 mM pH 7.5) were added. Then, KH_2_PO_4_ was added in order to reach pH ≈7.5, followed by 200 mg of CAL-B. It is worth noting that this amount represents a ≈8 fold decrease of used enzyme as compared to the corresponding small scale reaction (Table 2, entry 8). After 24 h, we were delighted to find that no enolester was detected by TLC monitoring, and after extraction and silica gel column chromatography (petroleum ether/EtOAc 9:1) a 68% isolated yield was obtained for the *S*-allyl-β-ketosulfide **2h**.

**Scheme 4:**
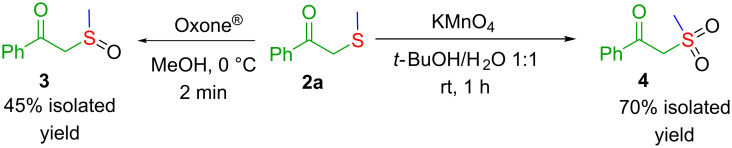
Selective oxidation of the β-ketosulfide **2a**.

The obtained β-ketosulfides can be chemoselectively converted into the corresponding sulfoxide/sulfone derivative [47–48]. It is well known that β-ketosulfoxides and β-ketosulfones are valuable moieties occurring in bioactive molecules, such as the immunosuppresor oxisurane [49], quinolone vasodilator flosequinan [50], and potential drugs for the treatment of diabetes [51]. As such, employing simple transformations, compound **2a** was conveniently oxidised in moderate to good isolated yields (45% ketosulfoxide **3**; 70% ketosulfone **4**, Scheme 4).

## Conclusion

In this work we have shown the development of a versatile chemoenzymatic methodology for the efficient preparation of β-ketosulfides avoiding the use of thiols. *Candida antarctica* lipase B resulted active in the presence of any tested cosolvent, regardless the buffer composition. Alternatively, PPL can be employed in the presence of DMSO as cosolvent and KPi buffer with good results. The steric congestion of the substrates resulted the main factor affecting the lipase activity, being the electronic nature of the substituents EDG/EWG, less important. The combination of the MCR and the lipase-catalysed hydrolysis can be carried out a in one-pot two-step fashion and afford the desired products in high isolated yield and high selectivity. A gram-scale experiment exemplifies the robustness of this methodology that can efficiently be employed in the further preparation of valuable ketosulfoxides and ketosulfones.

## Supporting Information

File 1 General procedures and NMR spectra.
