# Supplementary material for: Thiol-free chemoenzymatic synthesis of β-ketosulfides
Source: Beilstein J Org Chem. 2019 Feb 11;15:378–87. doi: 10.3762/bjoc.15.34 (PMC6404416; doi:10.3762/bjoc.15.34)

BEILSTEIN JOURNAL OF ORGANIC CHEMISTRY

Supporting Information

for

Thiol-free chemoenzymatic synthesis of β-ketosulfides

Adrián A. Heredia, Martín G. López-Vidal, Marcela Kurina-Sanz, Fabricio R. Bisogno and Alicia B. Peñéñory

Beilstein J. Org. Chem. **2019,** *15,* 378–387. doi:10.3762/bjoc.15.34

General procedures and NMR spectra

License and Terms: This is a supporting information file under the terms of the Creative Commons Attribution License (http://creativecommons.org/licenses/by/4.0). Please note that the reuse, redistribution and reproduction in particular requires that the authors and source are credited.
The license is subject to the *Beilstein Journal of Organic Chemistry* terms and conditions: (https://www.beilstein-journals.org/bjoc)

Table of contents

General methods... S2
General procedures.. S2
Spectroscopic characterization of synthesised compounds S4
References S9
^{1}H and ^{13}C spectra of all synthesised compounds.. S10

General methods

^{1}H and ^{13}C NMR spectra were recorded at 400.16 and 100.62 MHz, respectively, on a Bruker 400 spectrometer with $CDCl_3$ as a solvent. All spectra were reported in δ (ppm) relative to residual solvent signal [δ_H ($CHCl_3$) = 7.26 ppm]. Gas chromatographic analyses were performed on an Agilent 6890 with a flame-ionization detector, using a 30 m capillary column of a 0.32 mm × 0.25 μm film thickness, with a 5% phenylpolysiloxane phase. GC-MS analyses were conducted on Agilent 7890 employing a 30 m × 0.25 mm × 0.25 μm with a 5% phenylpolysiloxane phase column. HRMS spectra were recorded on a Premie orthogonal acceleration time-of-flight (oa-TOF) mass spectrometer. Ionization was achieved by electrospray and detection set on positive mode. The known β-ketosulfides displayed spectroscopic data in good agreement with those reported in literature.

Chemicals: All tested lipases are commercially available. Sodium phosphate diacid monohydrate, potassium phosphate dibasic heptahydrate, 'Tris' (tris(hydroxymethyl)aminomethane), 2-chloroacetophenone, allyl bromide, potassium thioacetate, Oxone®, sodium hydrogen sulfite, potassium permanganate and organic solvents were purchased from commercial sources (Merck, Sigma-Aldrich) and used as received. Ultrapure water from a Milli-Q station was used. The β-thioalkyl enol esters **1a–x** used as substrates in the lipase-catalysed hydrolysis were obtained following the methodology previously reported by our group.[1]

General procedures

General synthesis of β-alkylsulfide enol esters: β-alkylsulfide enol esters **1a**–**x** were synthesized according to previously reported procedure.[1] Briefly, the reactions were carried out in a 10 mL round-bottom flask, equipped with a magnetic bar. The flask was charged keeping the following addition order to ensure best yields: DMF (1.0 mL), thiocarboxylate (0.44 mmol), α-haloketone (0.44 mmol), alkyl or benzyl halide (generally 0.44 mmol, except for MeI, 0.88 mmol) and K_2CO_3 (0.88 mmol) were added and the mixture was stirred at room temperature for 5 h. Then, ethyl acetate (2 mL) and water (2 mL) were added and the mixture was stirred. The organic layer was separated, and the aqueous

layer was extracted with ethyl acetate (2 × 2 mL). The combined organic extract was dried over anhydrous Na_2SO_4 and the products were isolated by filtration through a silica gel pad from the crude reaction mixture.

Experimental procedure for the lipase-catalysed hydrolysis of β-thioalkyl enol esters 1a–x: In a 1.5 mL plastic conical-bottom vial, β-alkylsulfide enol ester (10 μL of a 600 mM DMSO stock solution) was added to 590 μL of phosphate buffer (0.1 M, pH 7.4). Then, CAL-B was added to the mixture according to Table 2. Reactions were shaken at 30 °C and 250 rpm for 24 h. Then, the reaction was stopped by extraction with ethyl acetate (0.6 mL). The organic layer was separated, and the aqueous layer was extracted with ethyl acetate (2 × 0.6 mL). The organic layers were pooled together and dried over anhydrous Na_2SO_4. Conversions were determined by GC analysis.

Experimental procedure for the semipreparative scale, one-pot, two-step preparation of 2: The reaction was carried out in a 10 mL screw-capped conical bottom plastic tube equipped with a magnetic stirring bar. 2-Haloacetophenone (0.25 mmol), 1.1 equiv of potassium thioacetate were dissolved in 500 μL of DMSO. Then, 2.0 equiv of K_2CO_3 and 1.1 equiv of alkyl halide were added and stirred at room temperature for 5 hours. Then, 9.5 mL of 50 mM phosphate buffer pH 7.5 and 100 mg of CAL-B were added. The tube was correctly closed and sealed, and the biocatalytic step took place in an orbital shaker (250 rpm) at 30 °C for 24 h. Finally, the reaction crude was extracted using of ethyl acetate (3 × 15 mL). The organic layer was dried with anhydrous Na_2SO_4. Corresponding compounds **2** were purified by column chromatography using a mixture of *n*-pentane/CH_2Cl_2 9:1 as the mobile phase. Compounds identities were corroborated by ^{1}H and ^{13}C NMR techniques and GC–MS.

Procedure for the synthesis of methyl phenacyl sulfoxide (3) from 2a: The reaction was carried out in a 5 mL round-bottom flask, equipped with a magnetic stirring bar. Phenacyl methyl sulfide **2a** (0.25 mmol) was dissolved in 2 mL of methanol. The mixture was placed into an ice bath until the temperature dropped to 0 °C. At this point, 3.0 equiv of $KHSO_5$ (50 mol % in Oxone®) were

added and stirred at this temperature for exactly 2 minutes. After this time, an excess of $NaHSO_3$ was added to quench the remaining oxidiser. The reaction crude was extracted using of ethyl acetate (3 × 15 mL). The pooled organic layers were dried with anhydrous Na_2SO_4. The corresponding sulfoxide thus obtained was purified by silica gel column chromatography using a mixture of *n*-pentane/CH_2Cl_2 9:1 as the mobile phase and its spectroscopic data agreed with those previously reported in the literature.

Procedure for the synthesis of methyl phenacyl sulfone (4) from 2a: The reaction was carried out in a 5 mL round-bottom flask, equipped with a magnetic bar. Phenacyl methyl sulfide **2a** (0.25 mmol) was dissolved in 2 mL of a mixture of *tert*-butanol/water 1:1. Potassium permanganate (1.2 equiv) was added and the mixture was stirring at room temperature for 1 h. After this time, an excess of $NaHSO_3$ was added to quench the remaining oxidiser. The reaction crude was extracted using of ethyl acetate (3 × 15 mL). The pooled organic layers were dried with anhydrous Na_2SO_4. The corresponding sulfone thus obtained was purified by silica gel column chromatography using a mixture of *n*-pentane/CH_2Cl_2 9:1 as the mobile phase and its spectroscopic data agreed with those previously reported in the literature.

Spectroscopic characterization of synthesised compounds

2-(Methylthio)-1-phenylethan-1-one (2a): [2] The corresponding compound was obtained as a yellow oil**.** ^{1}H NMR (400 MHz, $CDCl_3$): δ = 8.04 – 7.90 (m, 2H), 7.56 (dd, *J* = 10.7, 4.2 Hz, 1H), 7.45 (dd, *J* = 10.7, 4.2 Hz, 2H), 3.76 (s, 2H), 2.13 (s, 3H). ^{13}C NMR (100 MHz, $CDCl_3$): δ = 194.0; 135.2; 133.3; 128.7; 128.7; 39.0; 15.8. GC-MS (EI) m/z 166 (14) $[M]^+$, 105 (100), 77 (44), 51 (15).

2-(Methylthio)-1-(*p*-tolyl)ethan-1-one (2b): [2] The corresponding compound was obtained as a yellow oil. ^{1}H NMR (400 MHz, $CDCl_3$): δ = 7.88 (d, *J* = 8.2 Hz, 2H), 7.27 (d, *J* = 8.2 Hz, 2H), 3.74 (s, 2H), 2.42 (s, 4H), 2.14 (s, 3H). ^{13}C NMR (100 MHz, $CDCl_3$): δ = 194.0; 144.4; 132.9; 129.5; 129.0; 77.2; 39.2; 21.8; 16.0. GC-MS (EI) m/z 180 (8) $[M]^+$, 119 (100), 91 (44), 65 (14).

1-(4-Metoxyphenyl)-2-(methylthio)ethan-1-one (2c): [2] The corresponding compound was obtained as an orange oil. ^1H NMR (400 MHz, $CDCl_3$): δ = 7.96 (d, *J* = 8.9 Hz, 2H), 6.95 (d, *J* = 8.9 Hz, 2H), 3.88 (s, 3H), 3.72 (s, 2H), 2.14 (s, 3H). ^{13}C NMR (100 MHz, $CDCl_3$): δ = 193.1; 163.8; 131.2; 128.3; 114.0; 55.6; 39.0; 16.0. GC-MS (EI) m/z 196 (6) $[M]^+$, 150 (14), 135 (100), 107 (23), 92 (20), 77 (45).

4-(2-(Methylthio)acetyl)benzonitrile (2d): [3] The corresponding compound was obtained as an orange oil. ^1H NMR (400 MHz, DMSO-d_6): δ = 8.13 (d, *J* = 8.6 Hz, 2H), 8.01 (d, *J* = 8.6 Hz, 2H), 4.01 (s, 2H), 2.02 (s, 3H). ^{13}C NMR (100 MHz, DMSO-d_6): δ = 194.0; 139.0; 133.2; 129.7; 118.6; 115.7; 15.5. GC-MS (EI) m/z 191 (13) $[M]^+$, 145 (14), 130 (100), 102 (40), 75 (15), 61 (49).

1-(2-Bromophenyl)-2-(methylthio)ethan-1-one (2e): The corresponding compound was obtained as a yellow oil. ^1H NMR (400 MHz, $CDCl_3$): δ = 7.62 (dd, *J* = 7.9. 1.1 Hz, 1H), 7.47 (dd, *J* = 7.6, 1.8 Hz, 1H), 7.38 (td, *J* = 7.6, 1.2 Hz, 1H), 7.35 – 7.29 (m, 1H), 3.76 (s, 2H), 2.15 (s, 3H). ^{13}C NMR (100 MHz, $CDCl_3$): δ = 197.6; 140.0; 133.6; 131.8; 129.5; 127.3; 119.4; 42.5; 15.9. HRMS ESI calcd for $C_9H_9BrNaO_2S$ $[M+Na]^+$ (found as the corresponding sulfoxide): 282.9399, found 282.9401.

2-(Methylthio)-1-(4-nitrophenyl)ethan-1-one (2f): The corresponding compound was obtained as an dark orange oil. ^1H NMR (400 MHz, DMSO-d_6): δ = 8.34 (d, *J* = 8.9 Hz, 2H), 8.22 (d, *J* = 8.9 Hz, 2H), 4.04 (s, 2H), 2.03 (s, 3H). ^{13}C NMR (100 MHz, DMSO-d_6): δ = 193.7; 150.4; 140.5; 130.5; 124.3; 15.5. HRMS ESI $[M]^+$ calcd for $C_9H_{10}NO_3S$: 212.0376, found 212.0371.

2-((2-Hydroxyethyl)thio)-1-phenylethan-1-one (2g): [4] The corresponding compound was obtained as an dark orange oil. ^1H NMR (400 MHz, $CDCl_3$): δ = 8.02 – 7.94 (m, 2H), 7.62 – 7.56 (m, 1H), 7.52 – 7.44 (m, 2H), 3.88 (s, 2H), 3.77 (t, *J* = 5.8 Hz, 2H), 2.78 (t, *J* = 5.8 Hz, 2H). ^{13}C NMR (100 MHz, $CDCl_3$): δ = 195.0; 135.0; 133.5; 128.7; 128.6; 60.5; 36.9; 35.4. GC-MS (EI) m/z 178 (11) $[M-H_2O]^+$, 120 (8), 105 (100), 77 (44), 51 (14).

2-(Allylthio)-1-phenylethan-1-one (2h): [5] The corresponding compound was obtained as a pale green oil. ^{1}H NMR (400 MHz, $CDCl_3$): δ = 7.97 (dt, *J* = 8.5, 1.6 Hz, 2H), 7.62 – 7.54 (m, 1H), 7.51 – 7.44 (m, 2H), 5.78 (ddt, *J* = 17.1, 9.9, 7.2 Hz, 1H), 5.26 – 5.12 (m, 2H), 3.78 (s, 2H), 3.24 – 3.16 (m, 2H). ^{13}C NMR (100 MHz, $CDCl_3$): δ = 194.5; 135.5; 133.3; 133.0; 128.7 (2C); 118.5; 35.6; 34.8. GC-MS (EI) m/z 192 (4) $[M]^+$, 120 (41), 105 (100), 77 (46), 51 (14).

2-(Benzylthio)-1-phenylethan-1-one (2i): [5] The corresponding compound was obtained as a white solid. ^{1}H NMR (400 MHz, $CDCl_3$): δ = 7.94 – 7.91 (m, 2H), 7.64 – 7.52 (m, 1H), 7.51 – 7.41 (m, 2H), 7.39 – 7.20 (m, 5H), 3.76 (s, 2H), 3.67 (s, 2H). ^{13}C NMR (100 MHz, $CDCl_3$): δ = 194.5; 137.3; 135.5; 133.3; 129.3; 128.7; 128.7; 128.5; 127.3; 36.1; 35.9. GC-MS (EI) m/z 242 (6) $[M]^+$, 120 (67), 105 (100), 91 (48), 77 (49), 65 (14), 51 (14).

2-((2-Iodobenzyl)thio)-1-phenylethan-1-one (2j): The corresponding compound was obtained as a pale yellow solid. ^{1}H NMR (400 MHz, $CDCl_3$): δ = 7.97 (dt, *J* = 8.5, 1.6 Hz, 2H), 7.86 (dd, *J* = 7.9, 1.1 Hz, 1H), 7.63 – 7.56 (m, 1H), 7.49 – 7.43 (m, 3H), 7.31 (td, *J* = 7.5, 1.1 Hz, 1H), 6.95 (td, *J* = 7.7, 1.7 Hz, 1H), 3.87 (s, 2H), 3.74 (d, *J* = 2.8 Hz, 2H). ^{13}C NMR (100 MHz, $CDCl_3$): δ = 194.4; 140.0; 139.7; 135.3; 133.4; 130.6; 129.0; 128.8; 128.7; 128.3; 100.8; 41.1; 36.0. HRMS ESI calcd for $C_{15}H_{14}IOS$ $[M]^+$: 368.9805, found 368.9819.

2-(Butylthio)-1-phenylethan-1-one (2k): [5] The corresponding compound was obtained as a pale green oil. ^{1}H NMR (400 MHz, $CDCl_3$): δ = 8.04 – 7.93 (m, 2H), 7.63 – 7.52 (m, 1H), 7.53 – 7.43 (m, 2H), 3.78 (s, 2H), 2.57 (t, *J* = 7.3, 2H), 1.58 (quint, *J* = 7.3 Hz, 2H), 1.40 (quint, *J* = 7.3 Hz, 2H), 0.90 (t, *J* = 7.3 Hz, 3H). ^{13}C NMR (100 MHz, $CDCl_3$): δ = 194.6; 135.3; 133.3; 128.8; 128.6; 37.1; 32.1; 31.0; 21.9; 13.6. GC-MS (EI) m/z 208 (8) $[M]^+$, 120 (40), 105 (100), 61 (11), 77 (36), 51 (10).

1-((2-Iodobenzyl)thio)octan-2-one (2l): The corresponding compound was obtained as a colourless oil. ^{1}H NMR (400 MHz, $CDCl_3$): δ = 7.85 (dd, *J* = 7.9, 1.0 Hz, 1H), 7.36 (dd, *J* = 7.6, 1.7 Hz, 1H), 7.30 (td, *J* = 7.5, 1.2 Hz, 1H), 6.95 (td, *J* = 7.7, 1.7 Hz, 1H), 3.79 (s, 2H), 3.15 (s, 2H), 2.59 (t, *J* = 7.4 Hz, 2H), 1.58

(quint, J = 7.3 Hz, 2H), 1.31 – 1.28 (m, 6H), 0.89 (m, 3H). ^{13}C NMR (100 MHz, $CDCl_3$): δ = 206.0; 140.0; 139.8; 130.5; 129.0; 128.3; 100.6; 41.1; 40.6; 40.0; 31.6; 28.8; 23.9; 22.5; 14.0. HRMS ESI calcd for $C_{15}H_{22}IOS$ $[M]^+$: 377.0431, found 377.0442.

2-(Methylthio)-3,4-dihydronaphthalen-1(2*H*)-one (2m): [6] The corresponding compound was obtained as a colourless oil. ^{1}H NMR (400 MHz, $CDCl_3$): δ = 8.10 (dd, J = 7.8, 1.2 Hz, 1H), 7.47 (td, J = 7.5, 1.4 Hz, 1H), 7.33 (t, J = 7.6 Hz, 1H), 7.22 (d, J = 7.7 Hz, 1H), 3.50 (t, J = 4.2 Hz, 1H), 3.22 (ddd, J = 16.8, 11.8, 4.7 Hz, 1H), 2.84 (dt, J = 17.1, 4.2 Hz, 1H), 2.53 (ddt, J = 14.0, 11.8, 4.5 Hz, 1H), 2.33 (ddd, J = 13.9, 8.4, 4.3 Hz, 1H), 2.16 (s, 3H). ^{13}C NMR (100 MHz, $CDCl_3$): δ = 192.4; 142.8; 133.4; 130.8; 128.6; 128.2; 126.8; 50.1; 28.6; 25.9; 14.2. GC-MS (EI) m/z 192 (13) $[M]^+$, 146 (100), 131 (17), 115 (55), 90 (4), 63 (11).

(*Z*)-1-(Allylthio)prop-1-en-2-yl acetate (1v): The corresponding compound was obtained as an orange oil. ^{1}H NMR (400 MHz, $CDCl_3$):δ = 5.81 (ddt, J = 17.0, 9.9, 7.1 Hz, 1H), 5.51 (bs, 1H), 5.16 (m, 2H), 3.24 (d, J =7.0 Hz, 2H), 2.18 (s, 3H), 1.95 (s, 3H). ^{13}C NMR (100 MHz, $CDCl_3$): δ = 168.2; 145.0; 134.0; 117.7; 110.6; 36.1; 20.7; 19.8. HRMS ESI calcd for $C_8H_{12}O_2SNa$ $[M+Na]^+$: 195.0450, found: 195.0436.

1-(Allylthio)propan-2-one (2v): [7] The corresponding compound was obtained as a yellow oil. ^{1}H NMR (400 MHz, $CDCl_3$): δ = 5.72 (ddt, J = 16.0, 11.0, 7.3 Hz, 1H), 5.10 (m, 2H), 3.17 (s, 2H), 3.09 (d, J = 7.3 Hz,2H), 2.25 (s, 3H). ^{13}C NMR (100 MHz, $CDCl_3$): δ = 203.7; 132.9; 118.3; 40.3; 36.5; 29.6. GC-MS (EI) m/z 130 (39) $[M]^+$, 87 (100), 73 (79).

(*Z*)-1-(But-3-en-1-ylthio)prop-1-en-2-yl acetate (1w): The corresponding compound was obtained as an orange oil. ^{1}H NMR (400 MHz, $CDCl_3$): δ = 5.80 (m, 1H), 5.54 (s,1H), 5.06 (m, 2H), 2.68 (t, J = 7.5 Hz, 2H), 2.36 (m, 2H), 2.17 (s, 3H), 1.95 (s, 3H).^{13}C NMR (100 MHz, $CDCl_3$): δ = 168.1, 145.3, 136.1, 116.3, 111.7, 34.4, 32.9, 20.7, 19.8. HRMS ESI calcd for $C_9H_{14}O_2SNa$ $[M+Na]^+$: 209.0607, found: 209.0597.

1-(But-3-en-1-ylthio)propan-2-one (2w): The corresponding compound was obtained as a yellow oil. ^{1}H NMR (400 MHz, $CDCl_3$): δ = 5.81 (m, 1H), 5.08 (m, 2H), 3.48 (s, 2H), 2.78 (t, *J* = 7.4 Hz, 2H), 2.45 (t, *J* = 7.0 Hz, 2H), 2.31 (s, 3H). ^{13}C NMR (100 MHz, $CDCl_3$): δ = 203.2, 136.0, 116.7, 48.9, 37.8, 33.3, 28.5. HRMS ESI calcd for $C_7H_{13}OS$ $[M+H]^+$: 145.0682, found: 145.0647.

(*Z*)-1-(Benzylthio)prop-1-en-2-yl acetate (1x): The corresponding compound was obtained as a colourless oil. ^{1}H NMR (400 MHz, $CDCl_3$): δ = 7.29 (m, 5H), 5.49 (bq, *J* = 1.0 Hz, 1H), 3.82 (s, 2H), 2.16 (s, 3H), 1.91 (d, *J* = 0.9 Hz, 3H). ^{13}C NMR (100 MHz, $CDCl_3$): δ = 168.2, 145.3, 137.5, 128.9, 128.6, 127.3, 110.7, 37.6, 20.7, 19.8. HRMS ESI calcd for $C_{12}H_{14}O_2SNa$ $[M+Na]^+$: 245.0607, found: 245.0598.

1-(Benzylthio)propan-2-one (2x): [8] The corresponding compound was obtained as a colourless oil. ^{1}H NMR (400 MHz, $CDCl_3$): δ = 7.30 (m, 5H), 3.69 (s, 2H), 3.11 (s, 2H), 2.24 (s, 3H). ^{13}C NMR (100 MHz, $CDCl_3$): δ = 203.6, 137.2, 129.2, 128.6, 127.3, 40.8, 36.0, 28.0. GC-MS (EI) m/z 180 $[M]^+$, 123 (22),122 (11), 91 (100).

2-(Methylsulfinyl)-1-phenylethan-1-one (3): [9] The corresponding compound was obtained as white solid. **(3):** ^{1}H NMR (400 MHz, $CDCl_3$): δ = 7.99 – 7.97 (m, 2H), 7.64 (tt, *J* = 7.4, 1.5 Hz, 1H), 7.53 – 7.49 (m, 2H), 4.49 (d, *J* = 14.4, 1H), 4.31 (d, *J* = 14.4, 1H), 2.77 (s, 3H). ^{13}C NMR (100 MHz, $CDCl_3$): δ = 192.0; 136.0; 134.5; 129.0; 128.8; 62.1; 39.6. GC-MS (EI) m/z 182 (10) $[M]^+$, 136 (7), 105 (100), 77 (45), 51 (20).

2-(Methylsulfonyl)-1-phenylethan-1-one (4): [10] The corresponding compound was obtained as a white solid. ^{1}H NMR (400 MHz, $CDCl_3$): δ = 8.02 – 8.00 (m, 2H), 7.67 (bt, *J* = 7.4, 1H), 7.54 (bt, *J* = 7.8, 2H), 4.61 (s, 2H), 3.16 (s, 3H). ^{13}C NMR (100 MHz, $CDCl_3$): δ = 189.2; 135.6; 134.8; 129.3; 129.1; 61.3; 41.8. GC-MS (EI) m/z 198 (6) $[M]^+$, 105 (100), 77 (44), 51 (15).

References

1. Heredia, A. A.; Soria-Castro, S. M.; Bouchet, L. M.; Oksdath-Mansilla, G.; Barrionuevo, C. A.; Caminos, D. A.; Bisogno, F. R.; Argüello, J. E.; Peñéñory, A. B. *Org. Biomol. Chem.* **2014**, *12*, 6516-6526.
2. Su, Q.; Zhao, Z-J.; Xu, F.; Lou, P-C.; Zhang, K.; Xie, D-X.; Shi, L.; Cai, Q-Y.; Peng. Z-H.; An, D-L. *Eur. J. Org. Chem.* **2013**, 1551-1557.
3. Arisawa, M.; Suwa, K.; Yamaguchi, M. *Org. Lett.* **2009**, *11*, 625-627.
4. Fascione, M. A.; Adshead, S. J.; Mandal, P. K.; Kilner, C. A.; Leach, A. G.; Turnbull, W. B. *Chem. Eur. J.* **2012**, *18*, 2987-2997.
5. Yu, H.; Dong, D.; Ouyang, Y.; Wang, Y.; Liu, Q. *Synlett* **2007**, *1*, 151-155.
6. Arisawa, M.; Toriyama, F.; Yamaguchi, M. *Tetrahedron* **2011**, *67*, 2305-2312.
7. Sun, X.; Song, Z.; Li, H.; Sun, C. *Chem. Eur. J.* **2013**,*19*, 17589-17594.
8. Holland, H. L.; Brown, F.M.; Barrett, F.; French, J.; Johnson, D.V. *J. Ind. Microbiol. Biotechnol*. **2003**, *30*, 292–301.
9. Kunieda, N.; Fujiwara, Y.; Suzuki, A.; Kinoshita, M. *Phosphorus, Sulfur Silicon Relat. Elem.* **1983**, *16*, 223-232.
10. Pan, X-J.; Gao, J.; Yuan, G-Q. *Tetrahedron* **2015**, *71*, 5525-5530.

^{1}H and ^{13}C spectra of all synthesised compounds

^{1}H NMR 2-(methylthio)-1-phenylethan-1-one (**2a**)

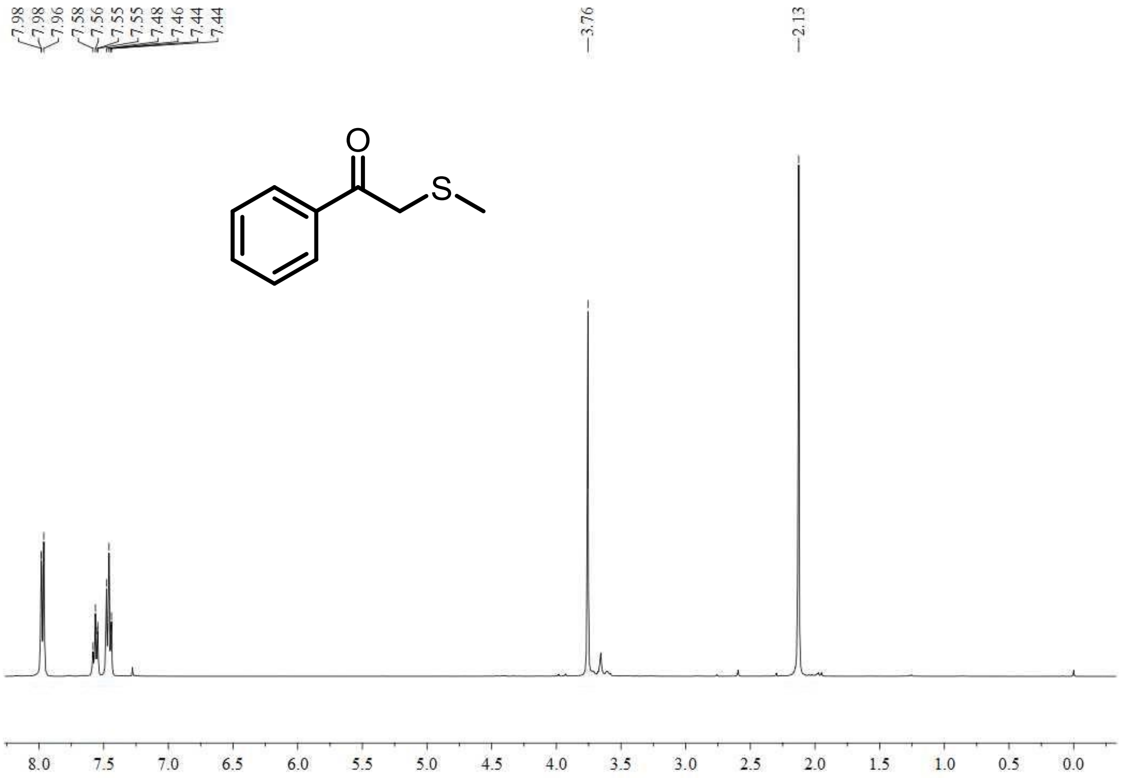

^{13}C NMR 2-(methylthio)-1-phenylethan-1-one (**2a**)

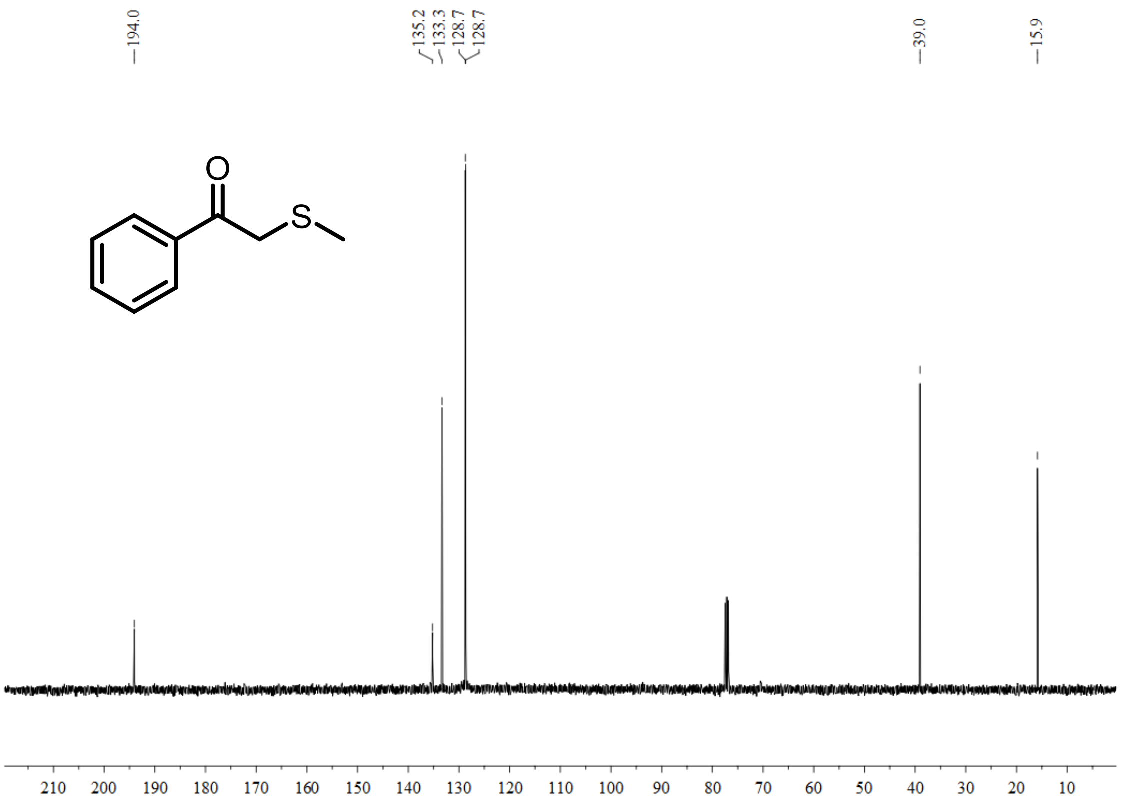

^{1}H NMR 2-(methylthio)-1-(p-tolyl)ethan-1-one (**2b**)

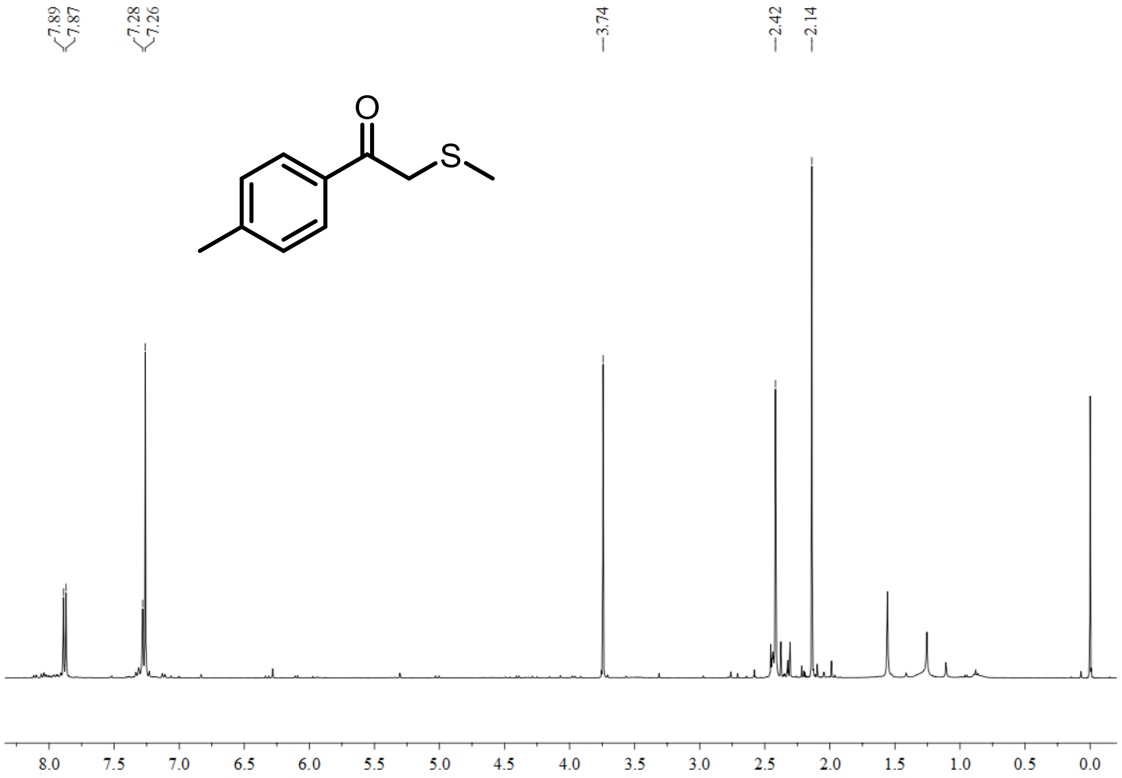

^{13}C NMR 2-(methylthio)-1-(p-tolyl)ethan-1-one (**2b**)

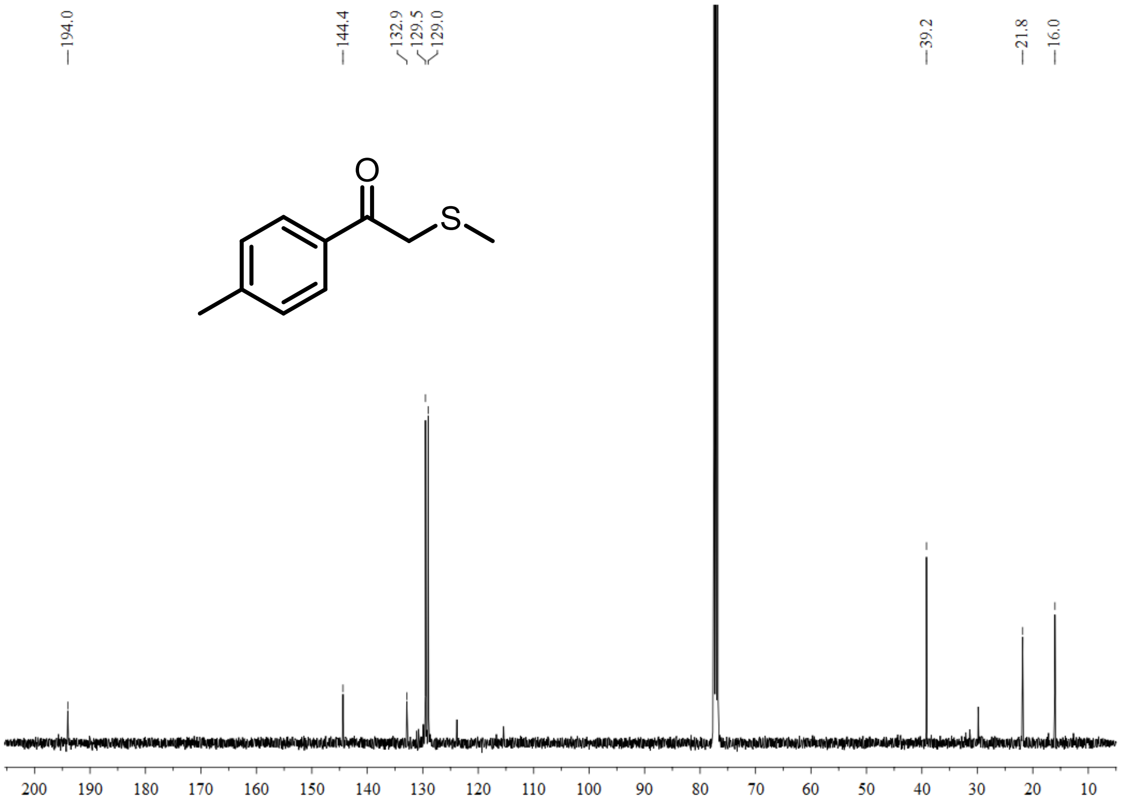

^{1}H NMR 1-(4-metoxyphenyl)-2-(methylthio)ethan-1-one (**2c**)

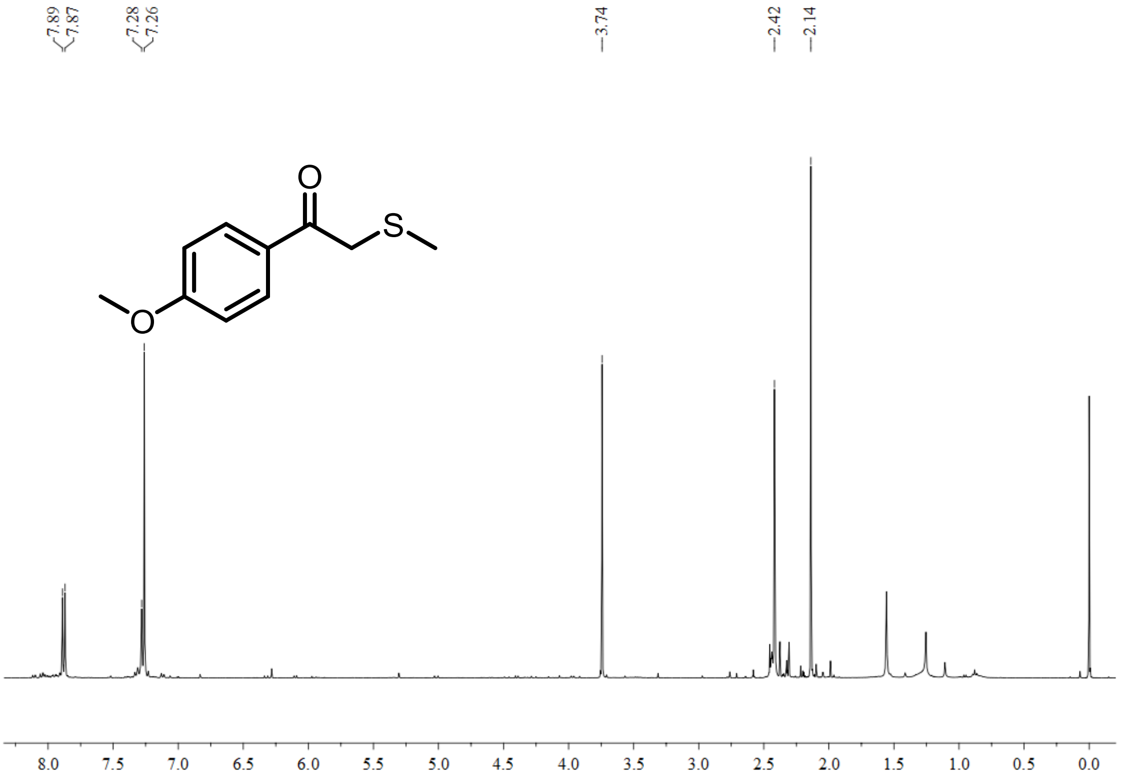

^{13}C NMR 1-(4-metoxyphenyl)-2-(methylthio)ethan-1-one (**2c**)

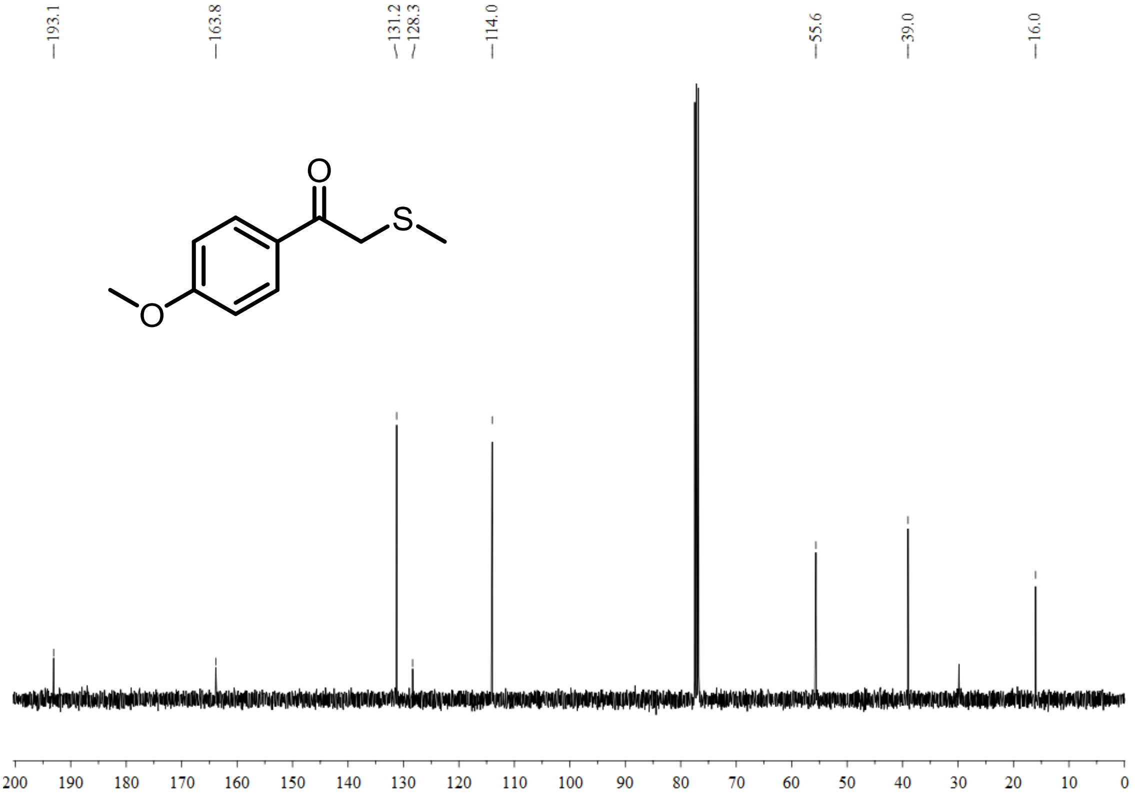

^{1}H NMR 4-(2-(methylthio)acetyl)benzonitrile (**2d**)

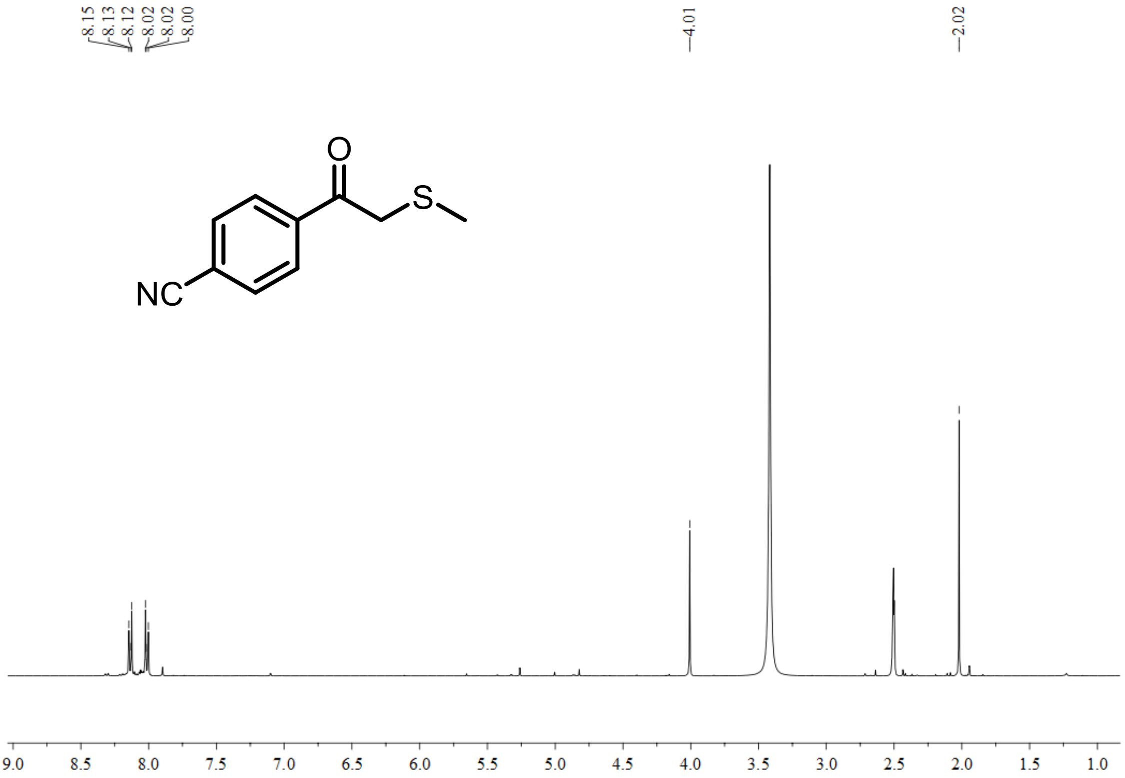

^{13}C NMR 4-(2-(methylthio)acetyl)benzonitrile (**2d**)

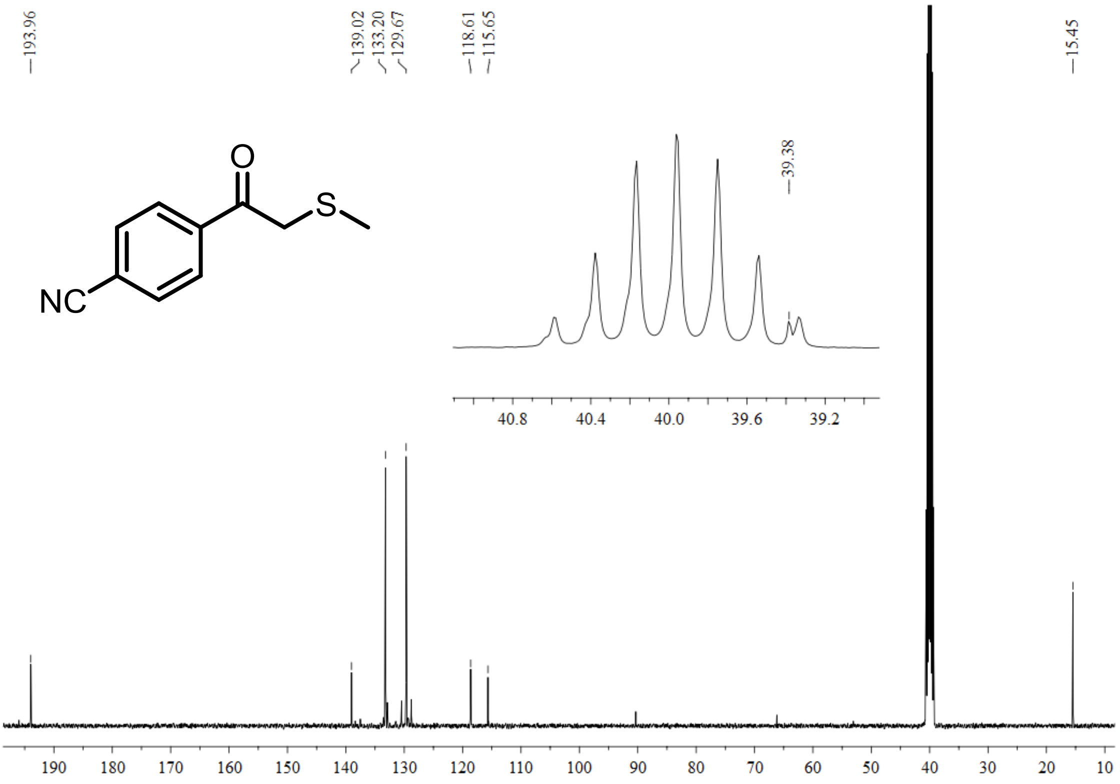

^{1}H NMR 1-(2-bromophenyl)-2-(methylthio)ethan-1-one (**2e**)

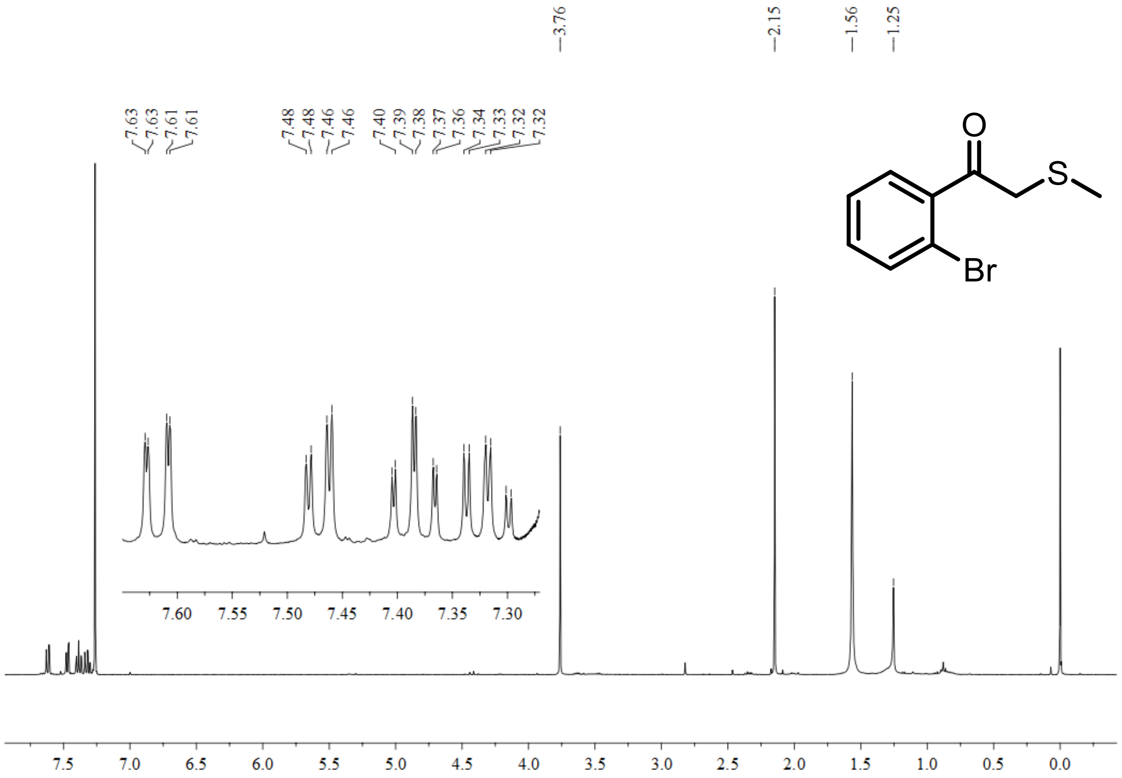

^{13}C NMR 1-(2-bromophenyl)-2-(methylthio)ethan-1-one (**2e**)

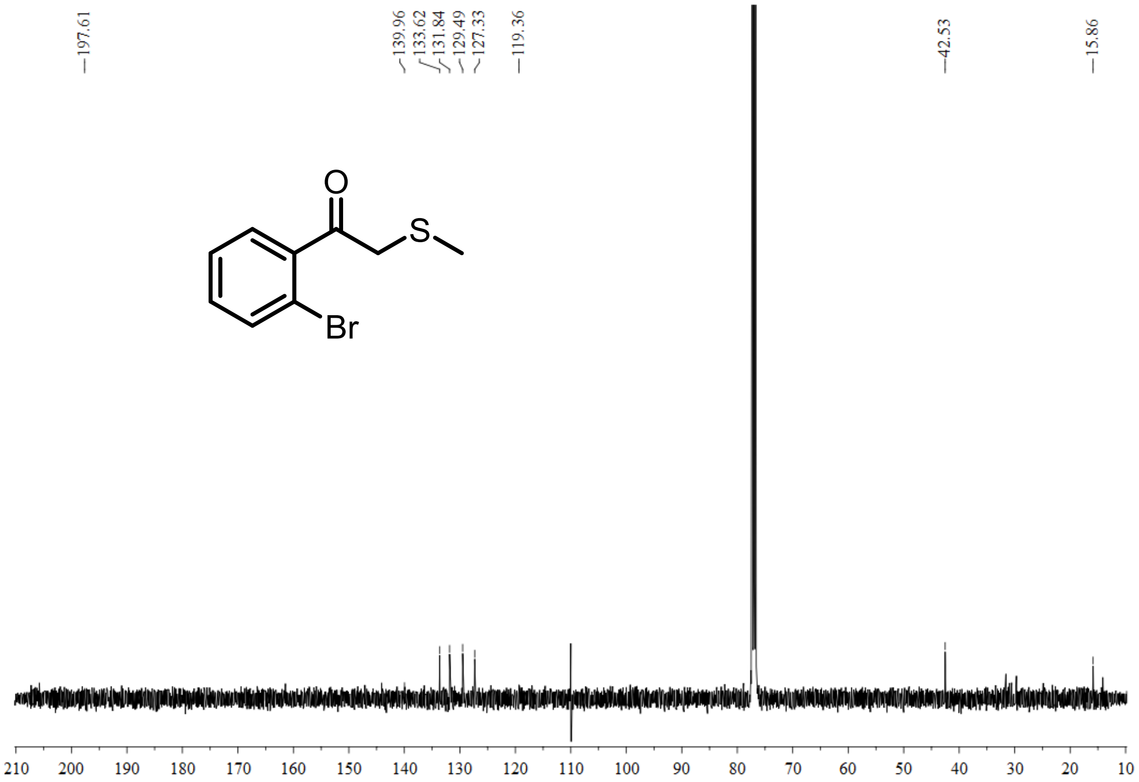

^{1}H-^{13}C HMBC 1-(2-bromophenyl)-2-(methylthio)ethan-1-one (**2e**)

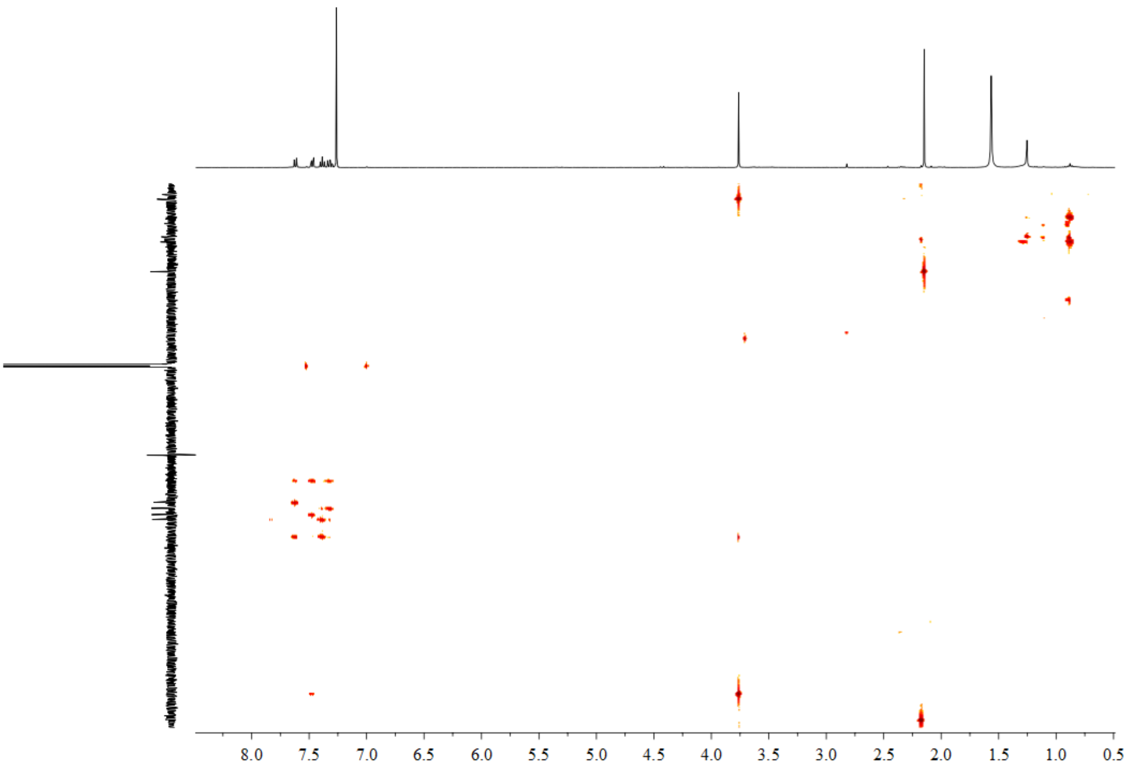

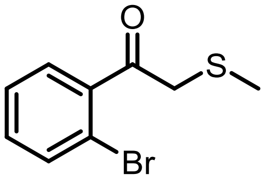

^{1}H NMR 2-(methylthio)-1-(4-nitrophenyl)ethan-1-one (**2f**)

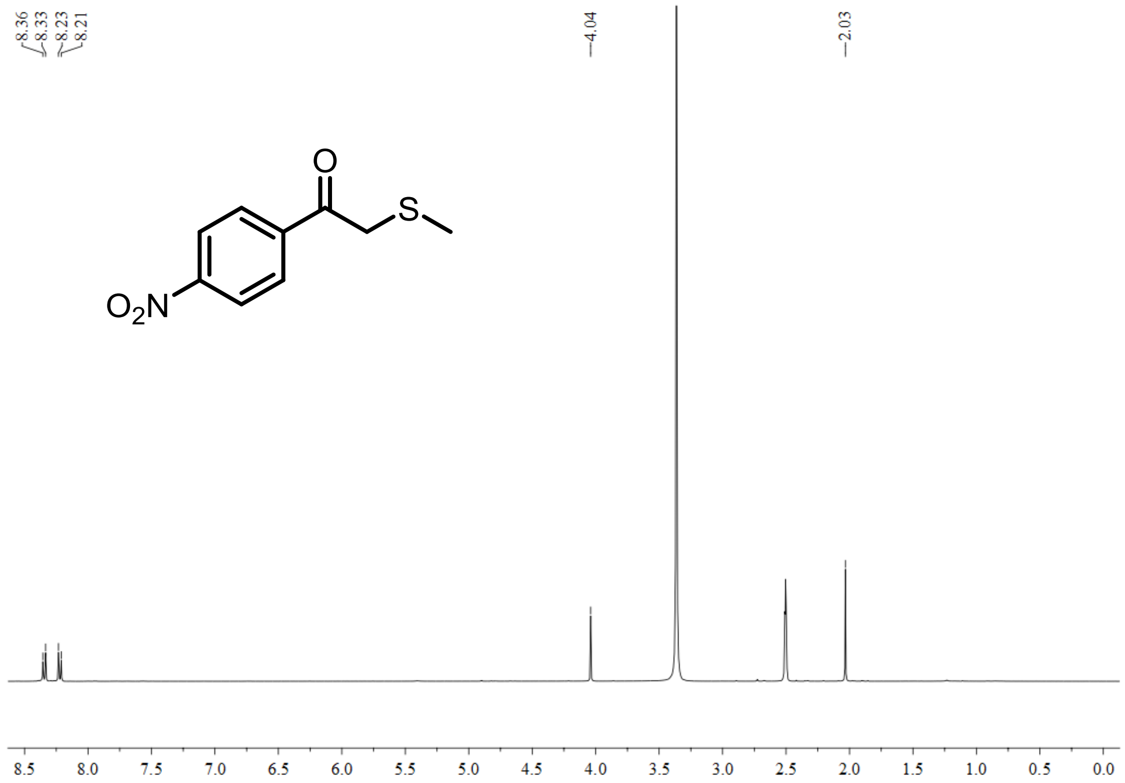

^{13}C NMR 2-(methylthio)-1-(4-nitrophenyl)ethan-1-one (**2f**)

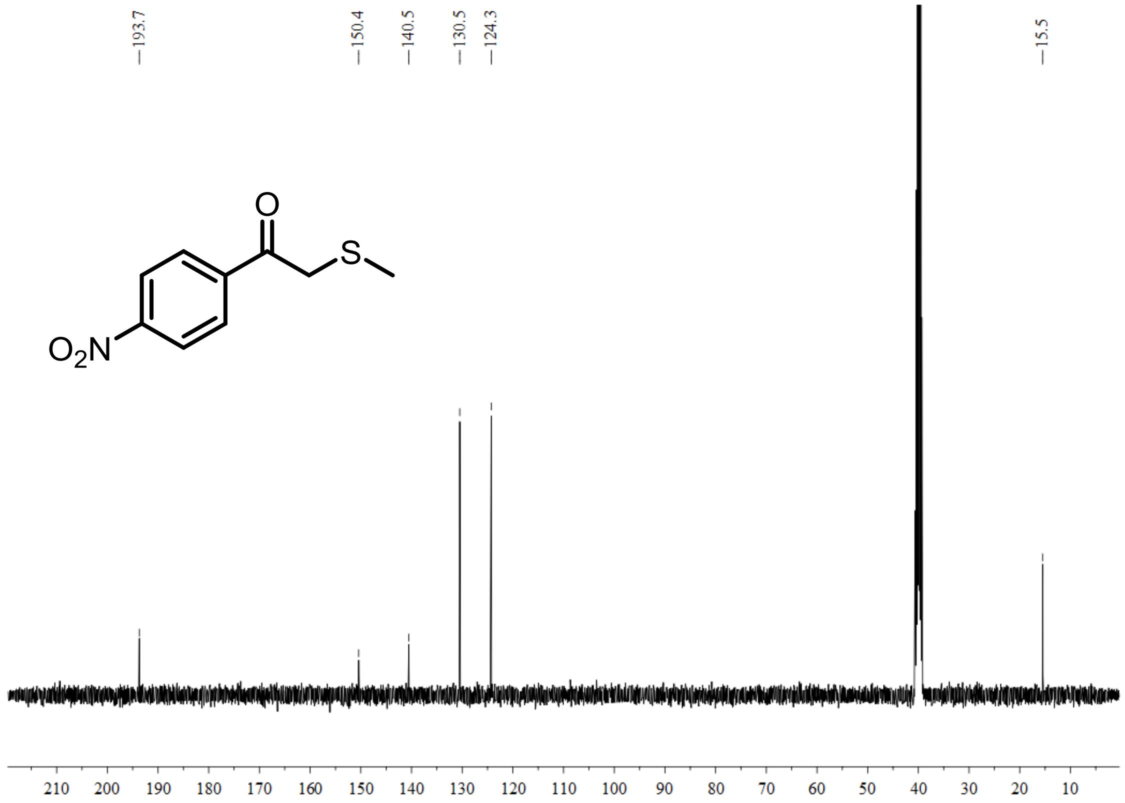

^1H-^{13}C HSQC 2-(methylthio)-1-(4-nitrophenyl)ethan-1-one (**2f**)

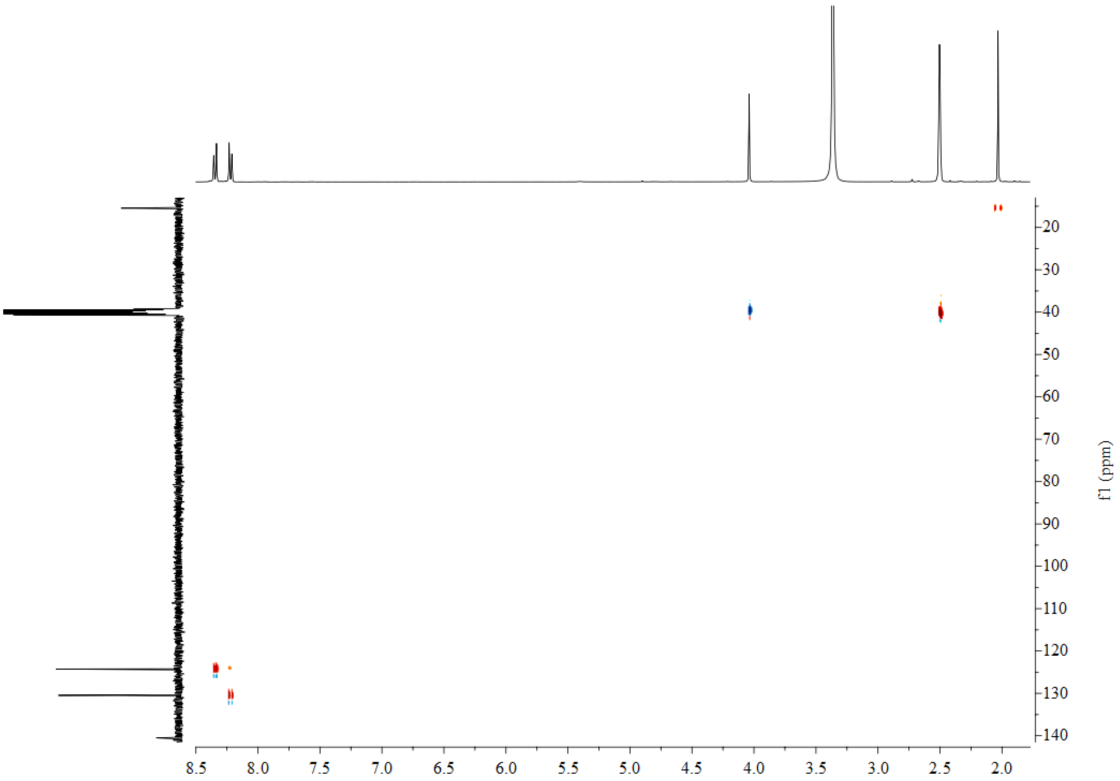

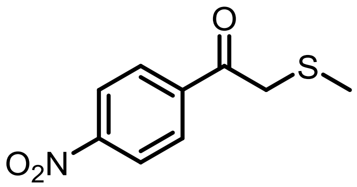

^{1}H NMR 2-((2-hydroxyethyl)thio)-1-phenylethan-1-one (**2g**)

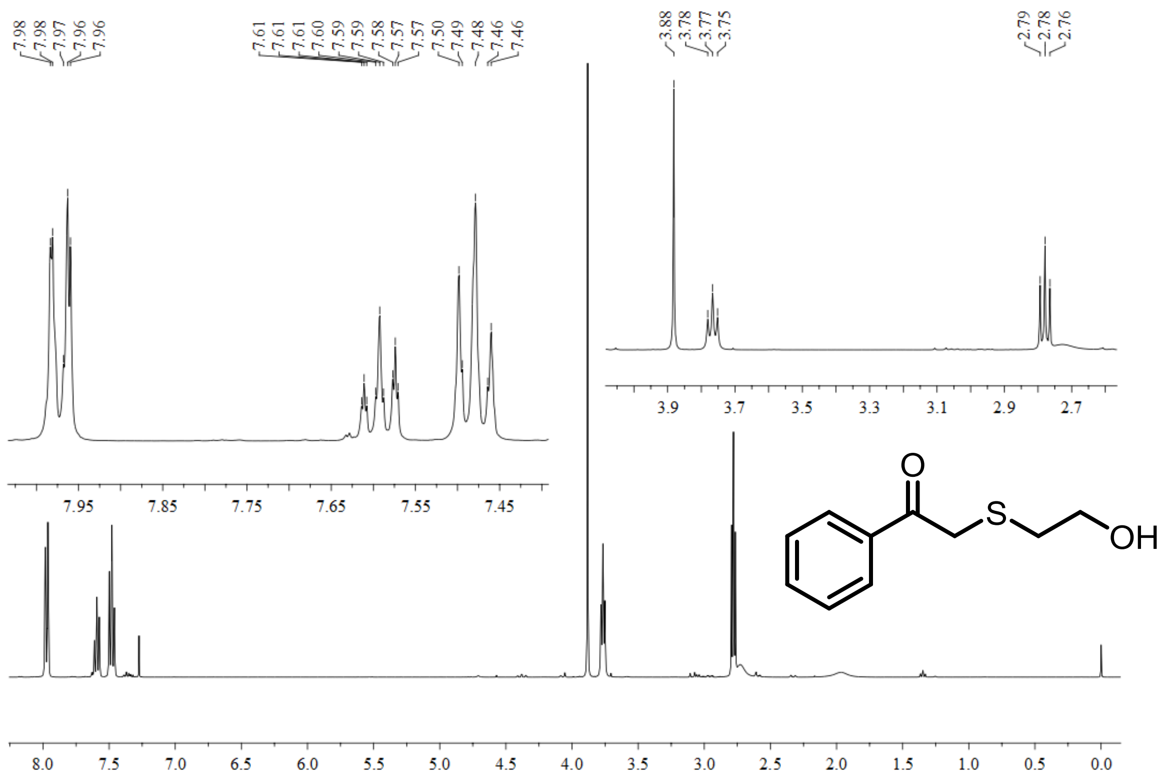

^{13}C NMR 2-((2-hydroxyethyl)thio)-1-phenylethan-1-one (**2g**)

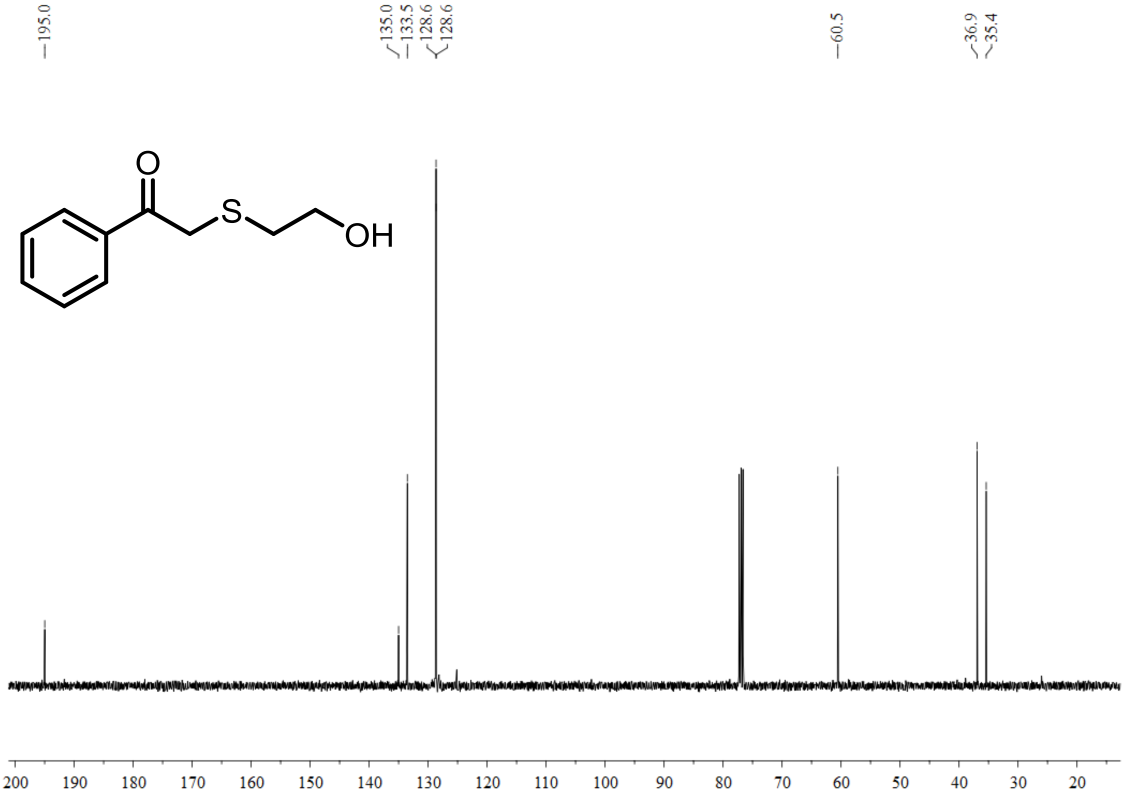

^{1}H NMR 2-(allylthio)-1-phenylethan-1-one (**2h**)

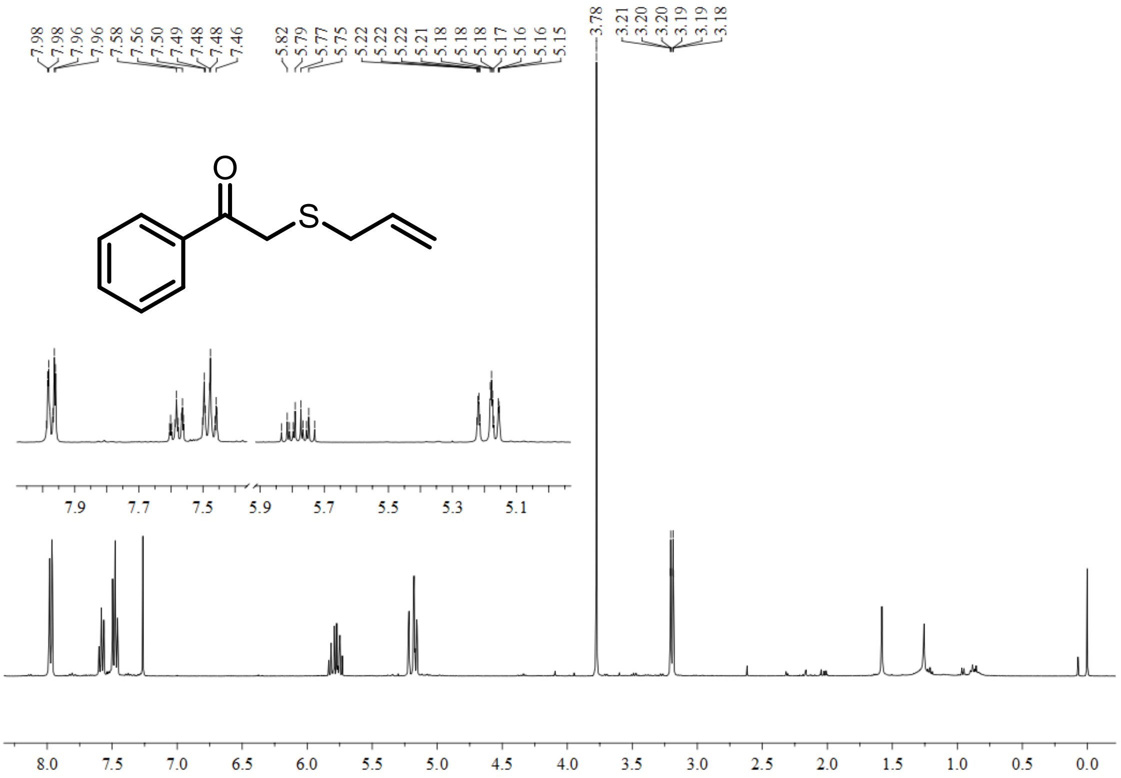

^{13}C NMR 2-(allylthio)-1-phenylethan-1-one (**2h**)

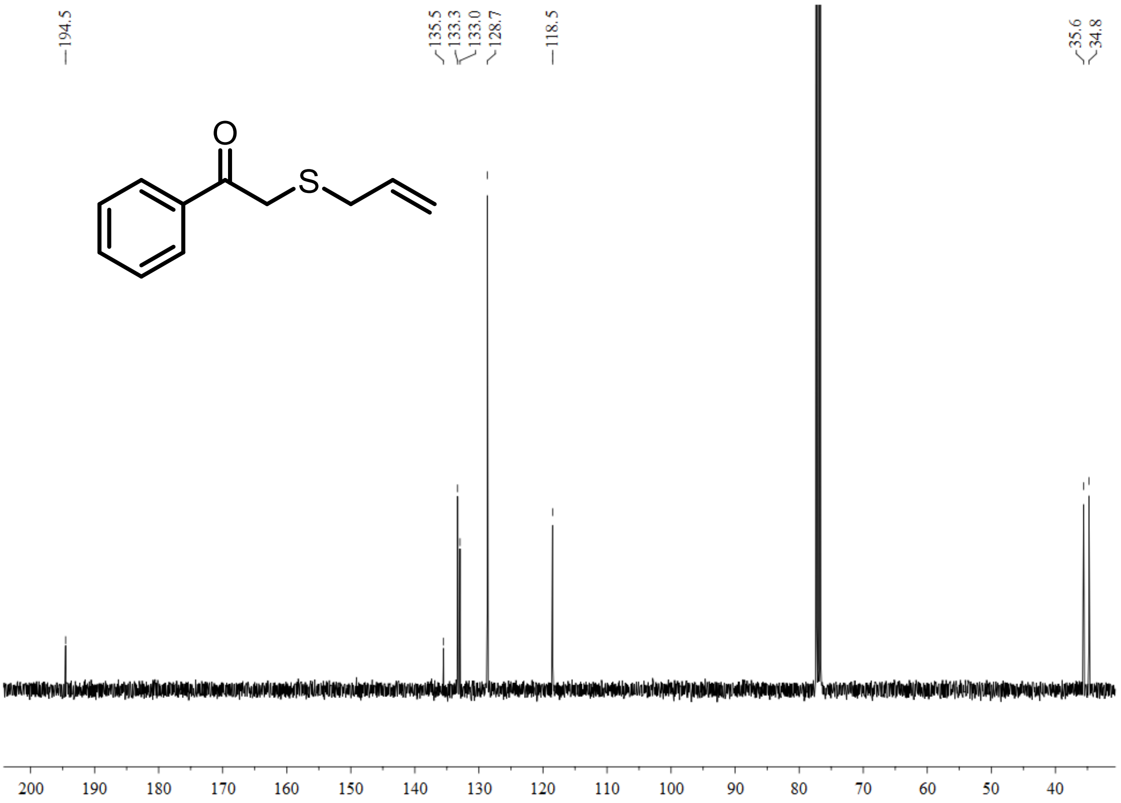

^{1}H-^{13}C HSQC 2-(allylthio)-1-phenylethan-1-one (**2h**)

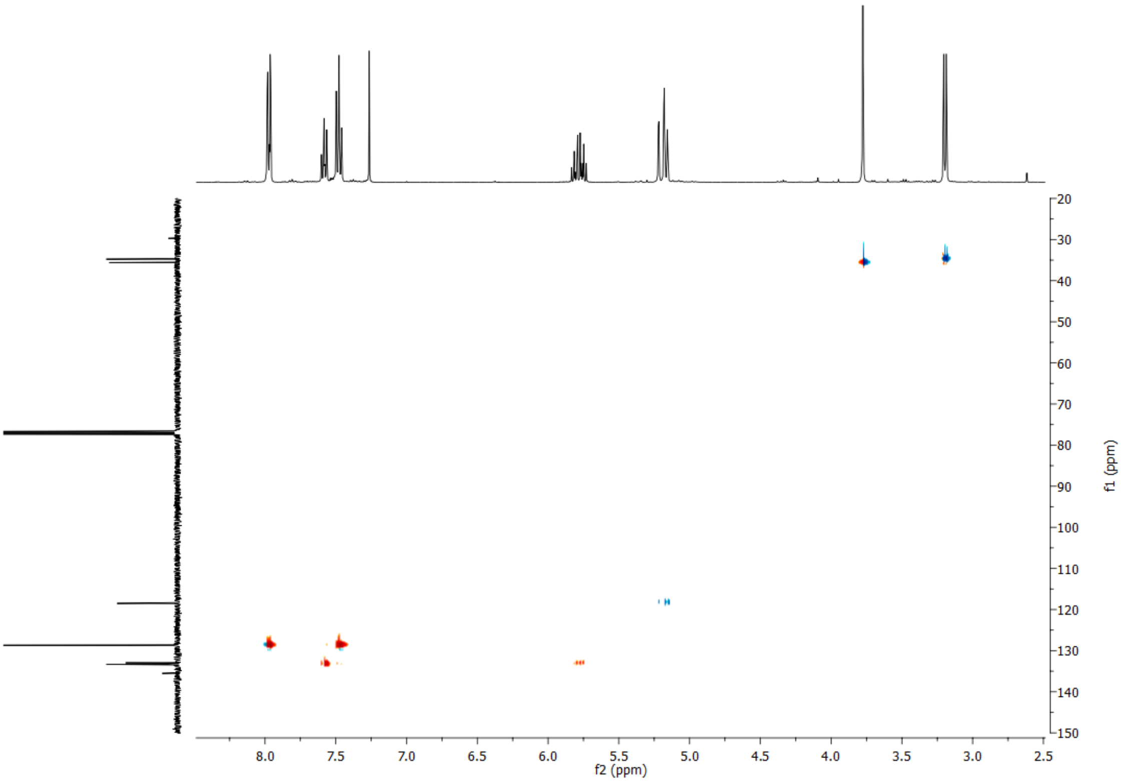

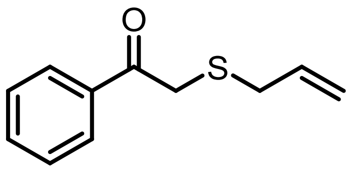

^{1}H NMR 2-(benzylthio)-1-phenylethan-1-one (**2i**)

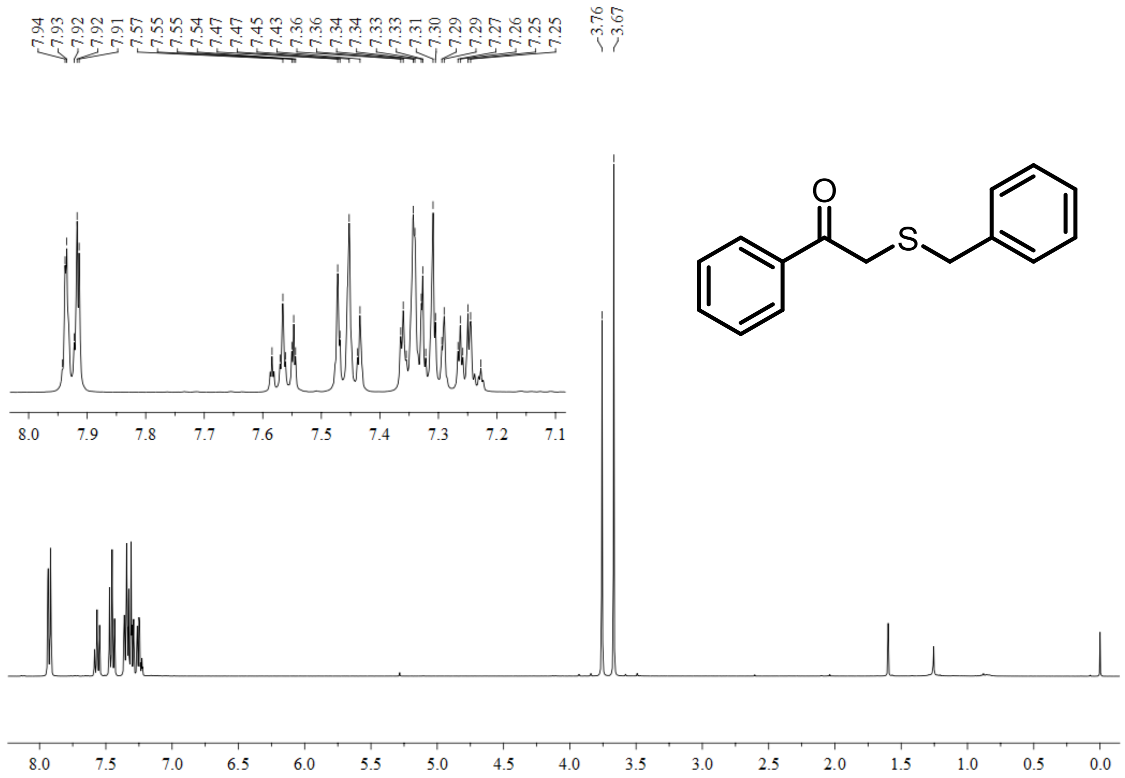

^{13}C NMR 2-(benzylthio)-1-phenylethan-1-one (**2i**)

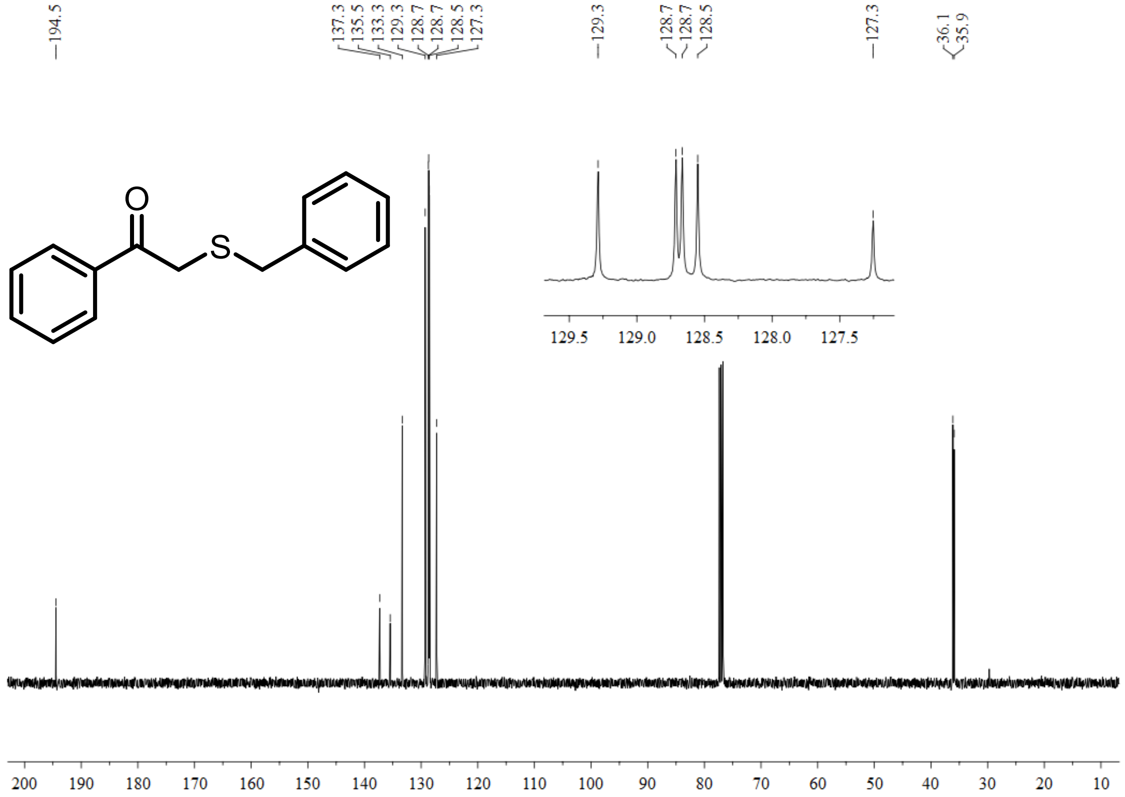

^{1}H NMR 2-((2-iodobenzyl)thio)-1-phenylethan-1-one (**2j**)

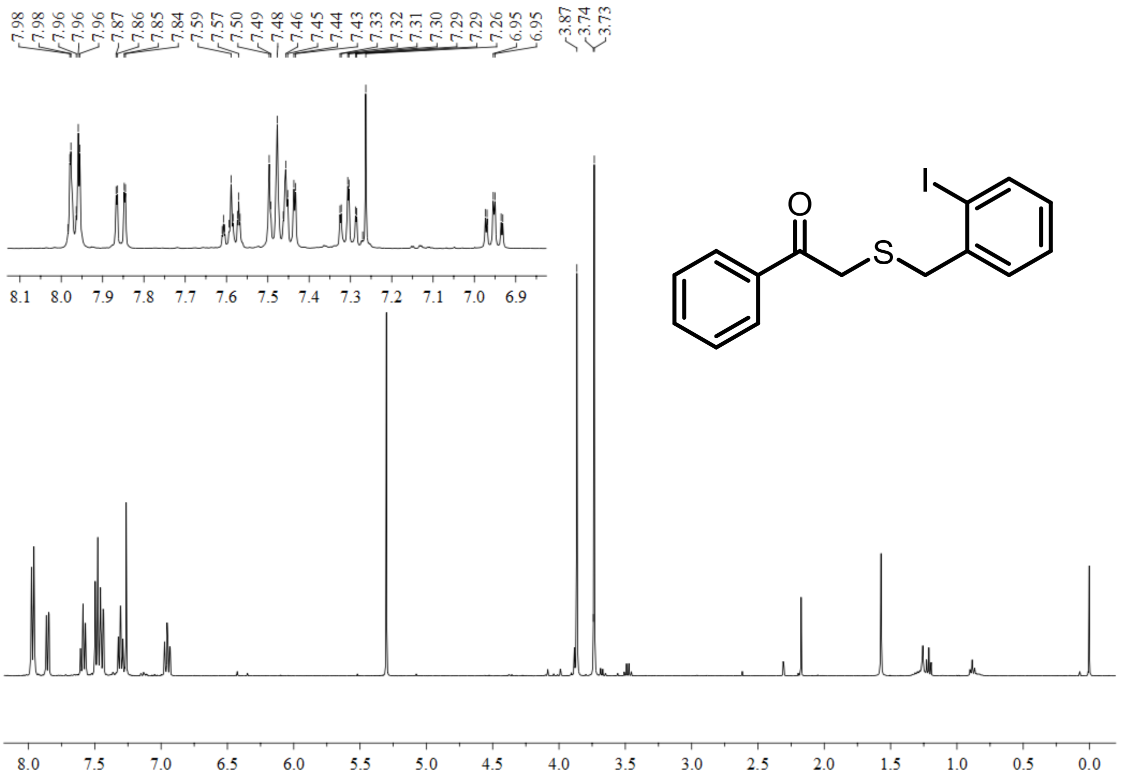

^{13}C NMR 2-((2-iodobenzyl)thio)-1-phenylethan-1-one (**2j**)

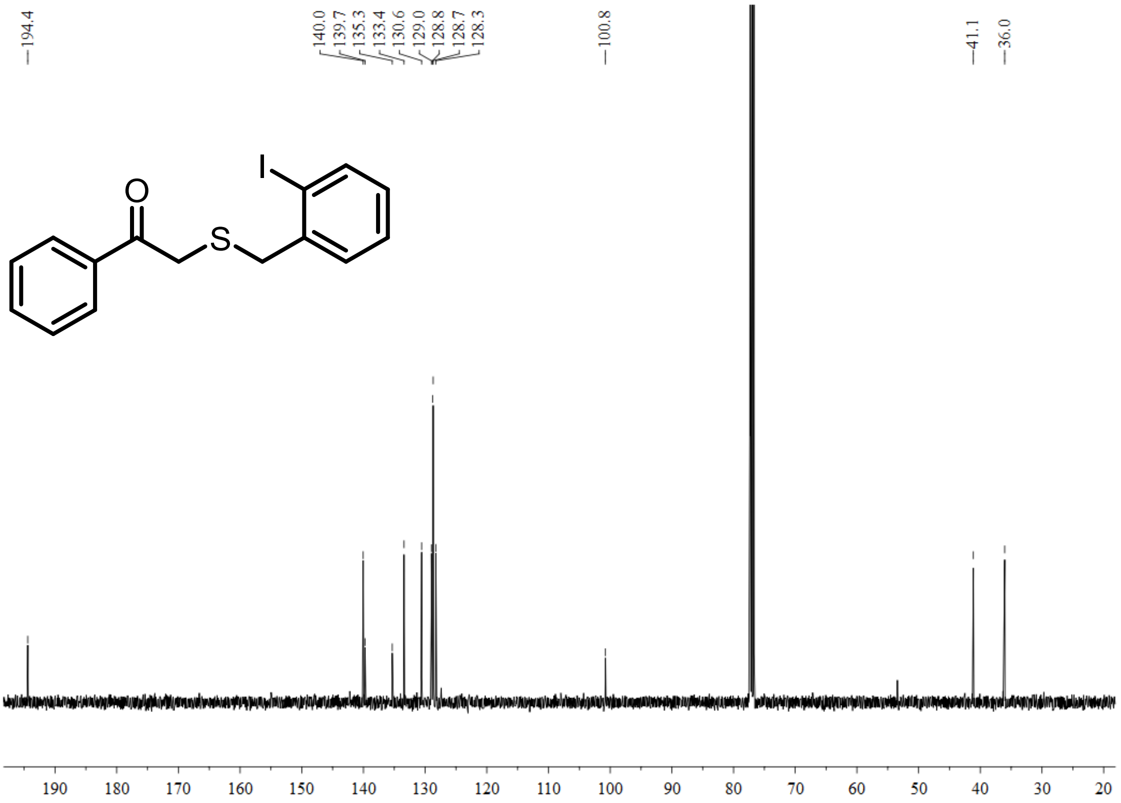

^{1}H NMR 2-(butylthio)-1-phenylethan-1-one (**2k**)

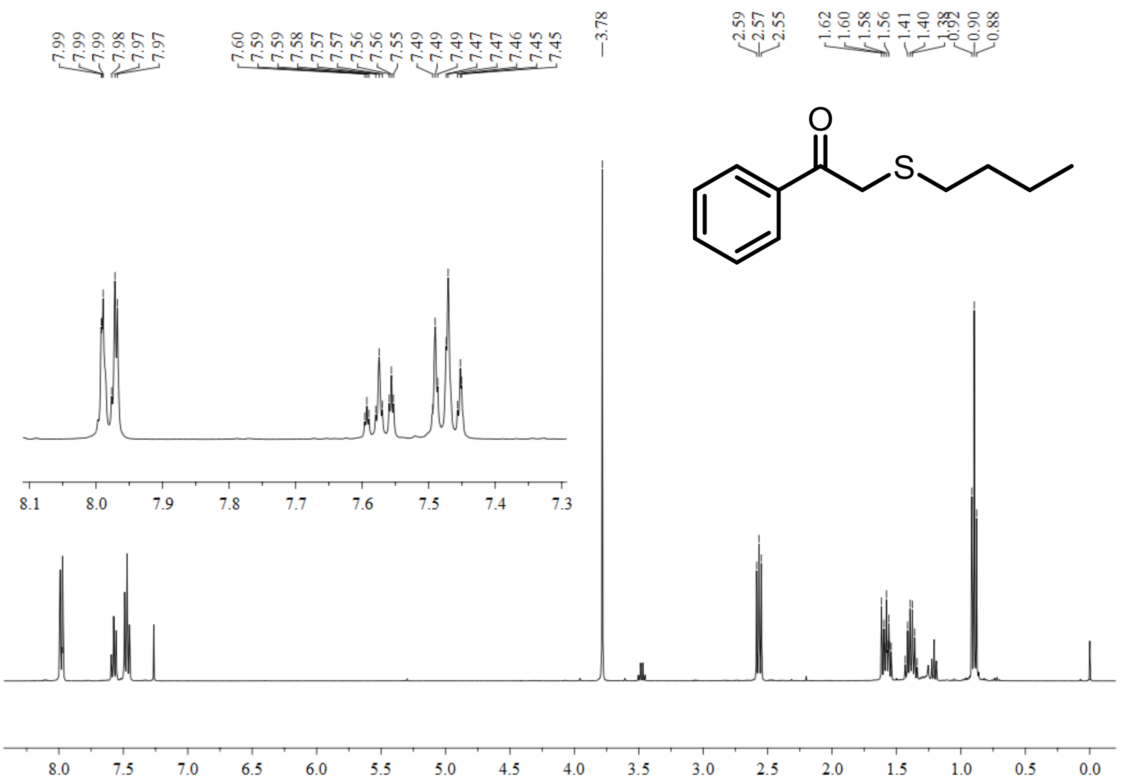

^{13}C NMR 2-(butylthio)-1-phenylethan-1-one (**2k**)

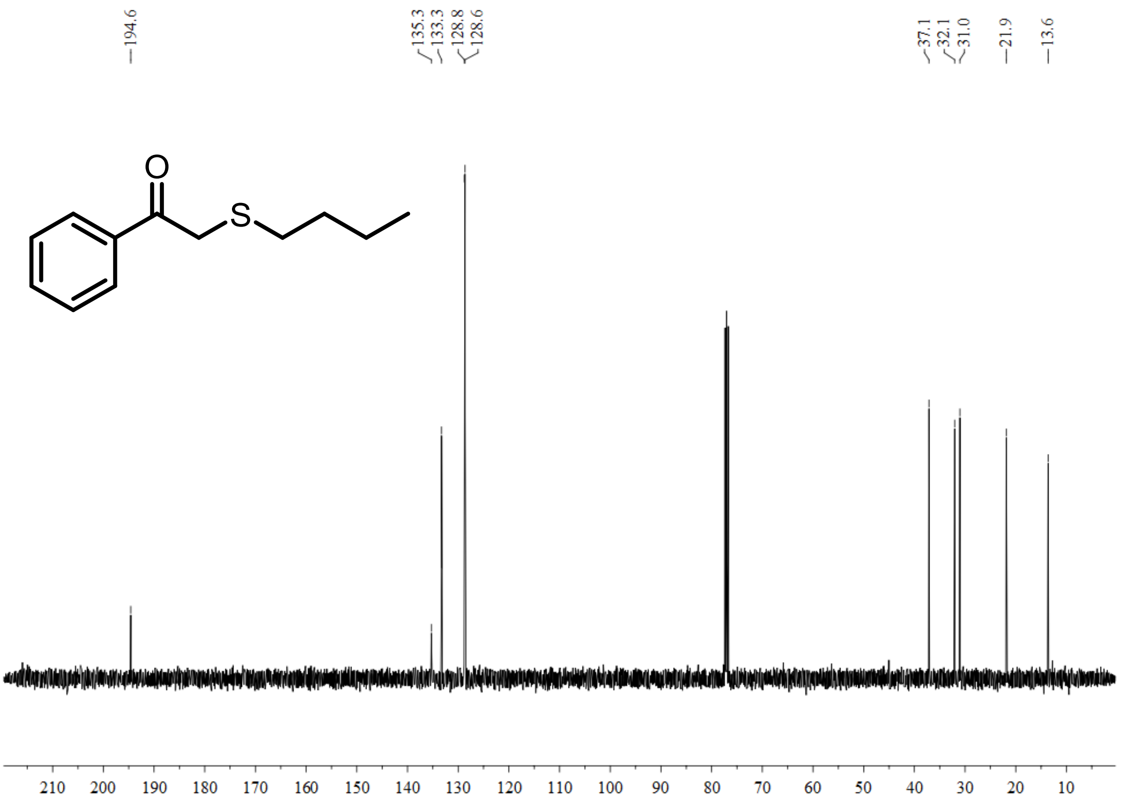

^{1}H NMR 1-((2-iodobenzyl)thio)octan-2-one (**2h**)

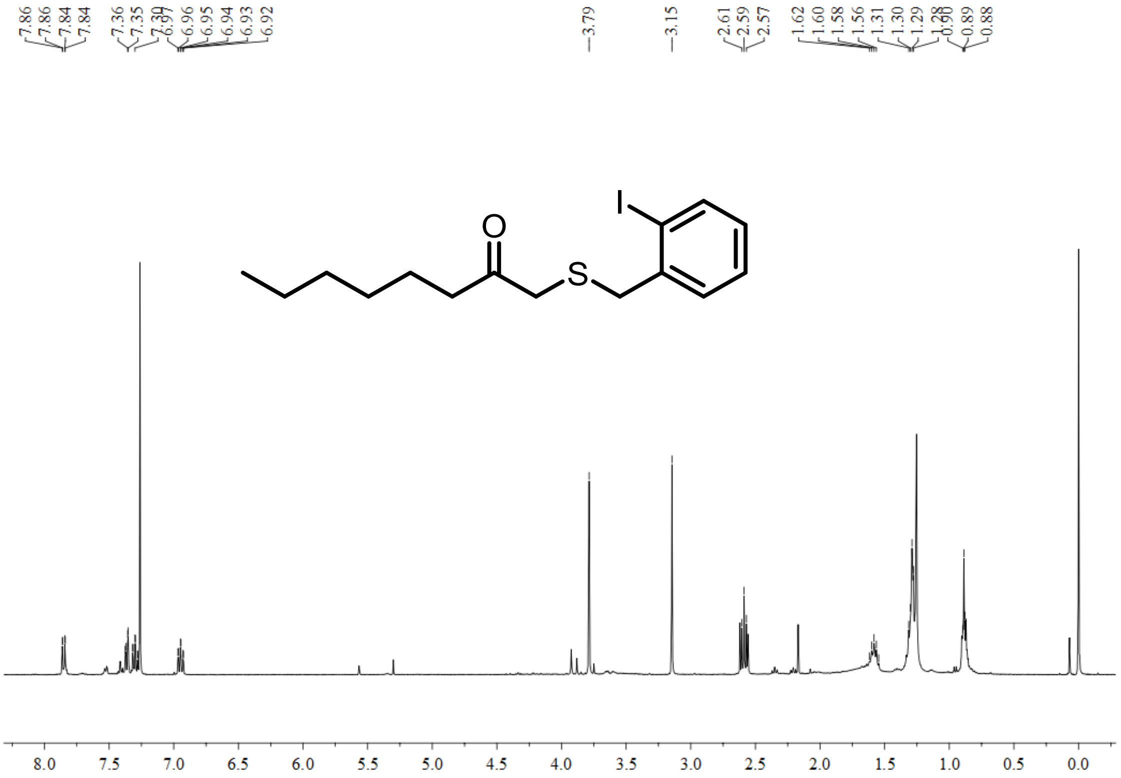

^{13}C NMR 1-((2-iodobenzyl)thio)octan-2-one (**2h**)

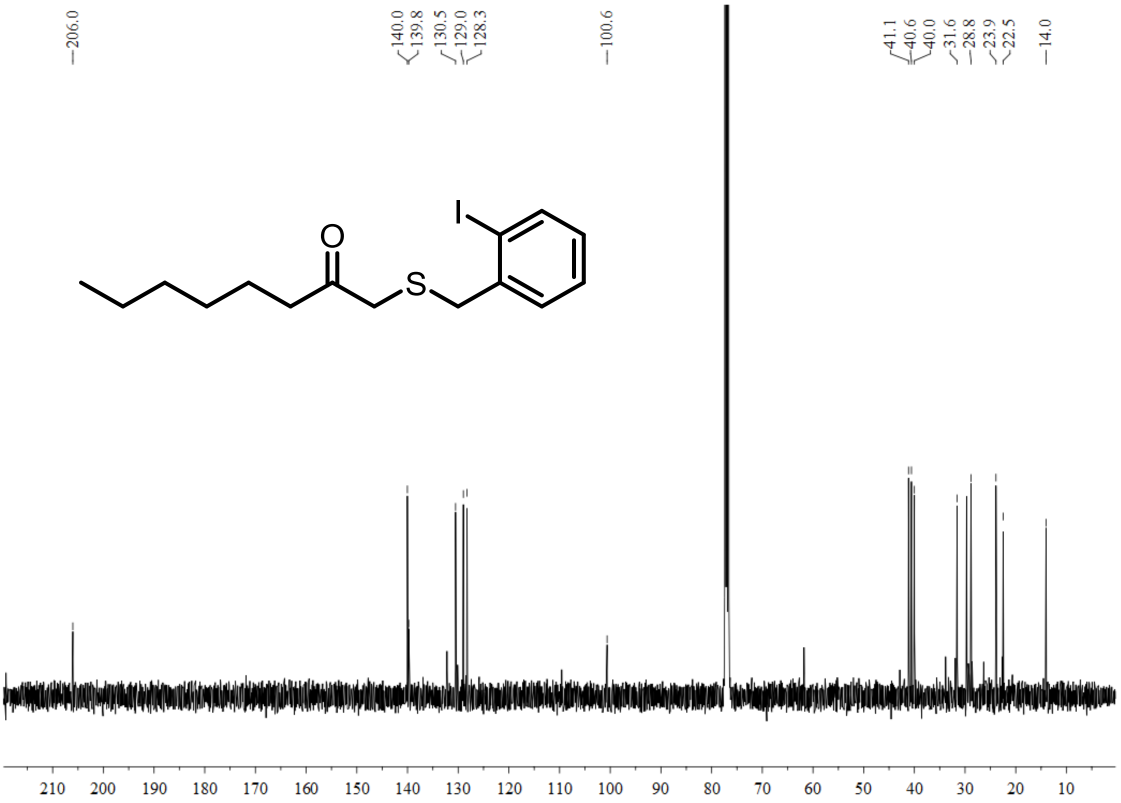

^{1}H NMR 2-(methylthio)-3,4-dihydronaphthalen-1(2H)-one (**2m**)

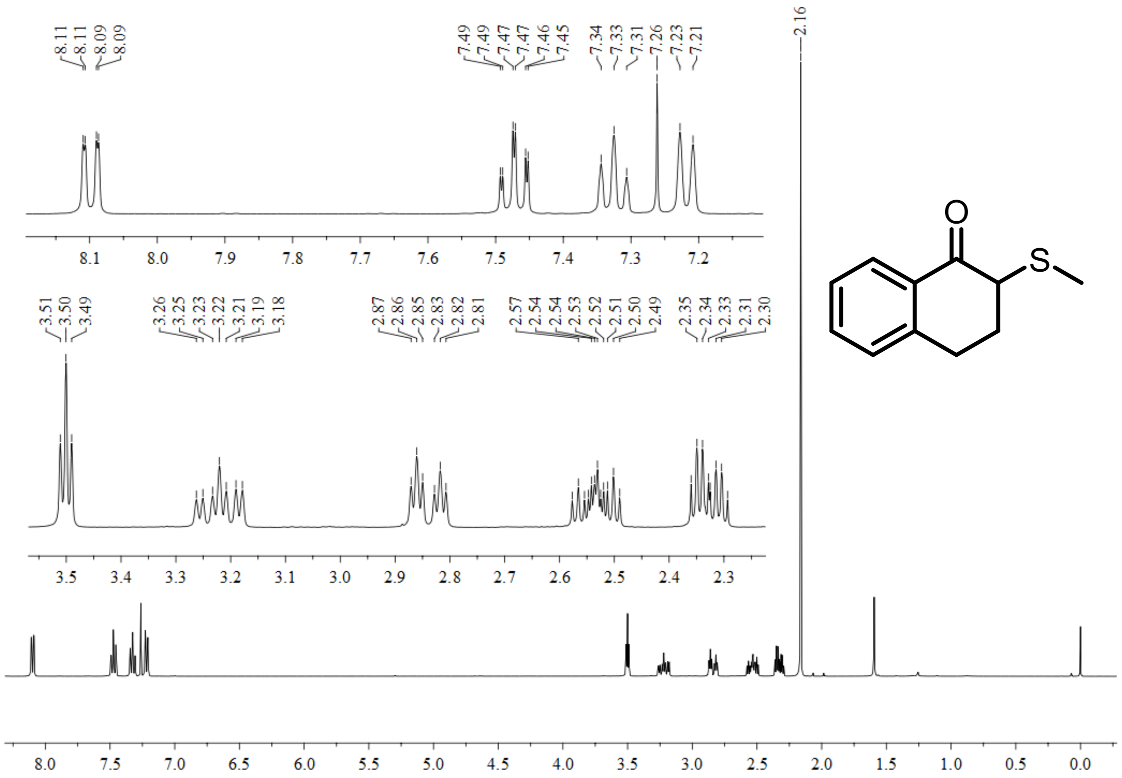

^{13}C NMR 2-(methylthio)-3,4-dihydronaphthalen-1(2H)-one (**2m**)

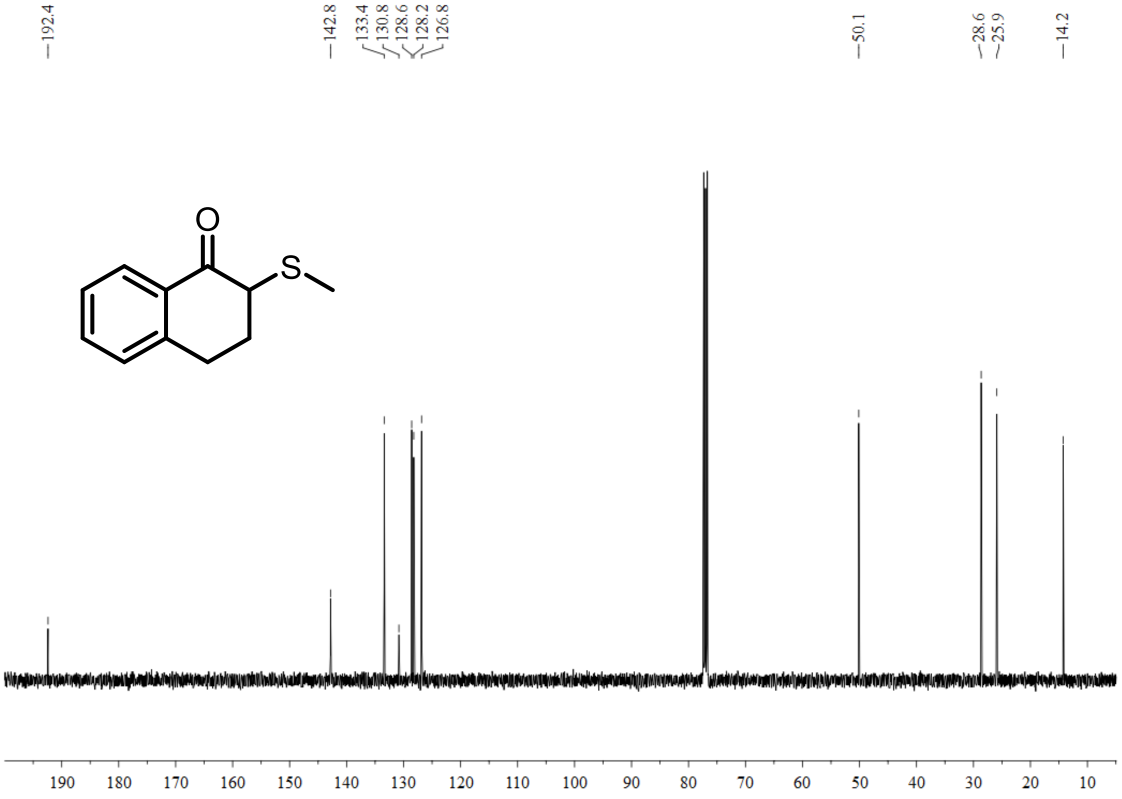

^{1}H NMR (Z)-1-(allylthio)prop-1-en-2-yl acetate (**1v**)

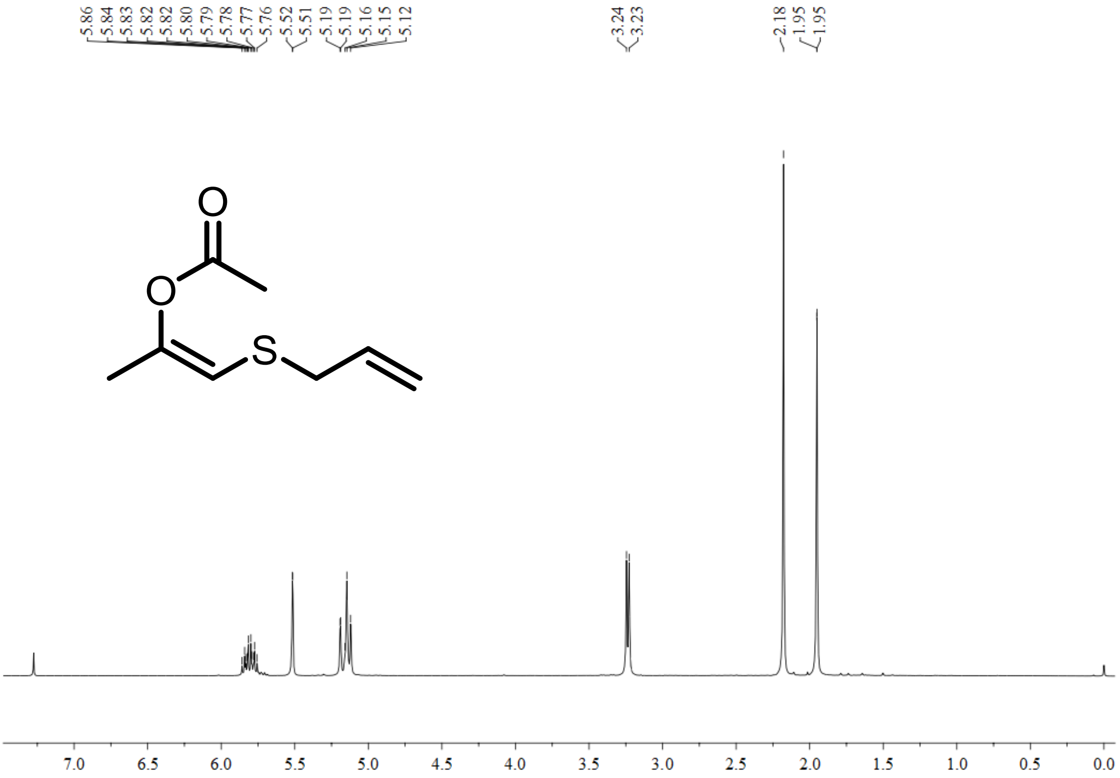

^{13}C NMR (Z)-1-(allylthio)prop-1-en-2-yl acetate (**1v**)

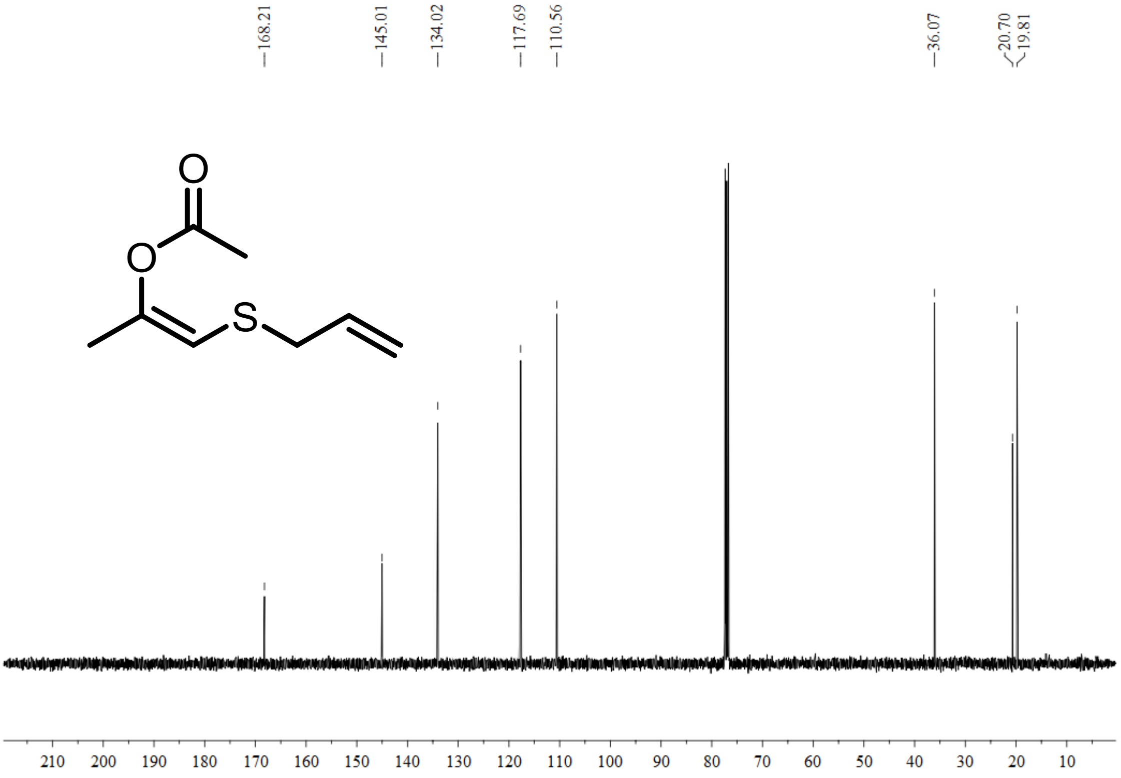

^{1}H NMR 1-(allylthio)propan-2-one (**2v**)

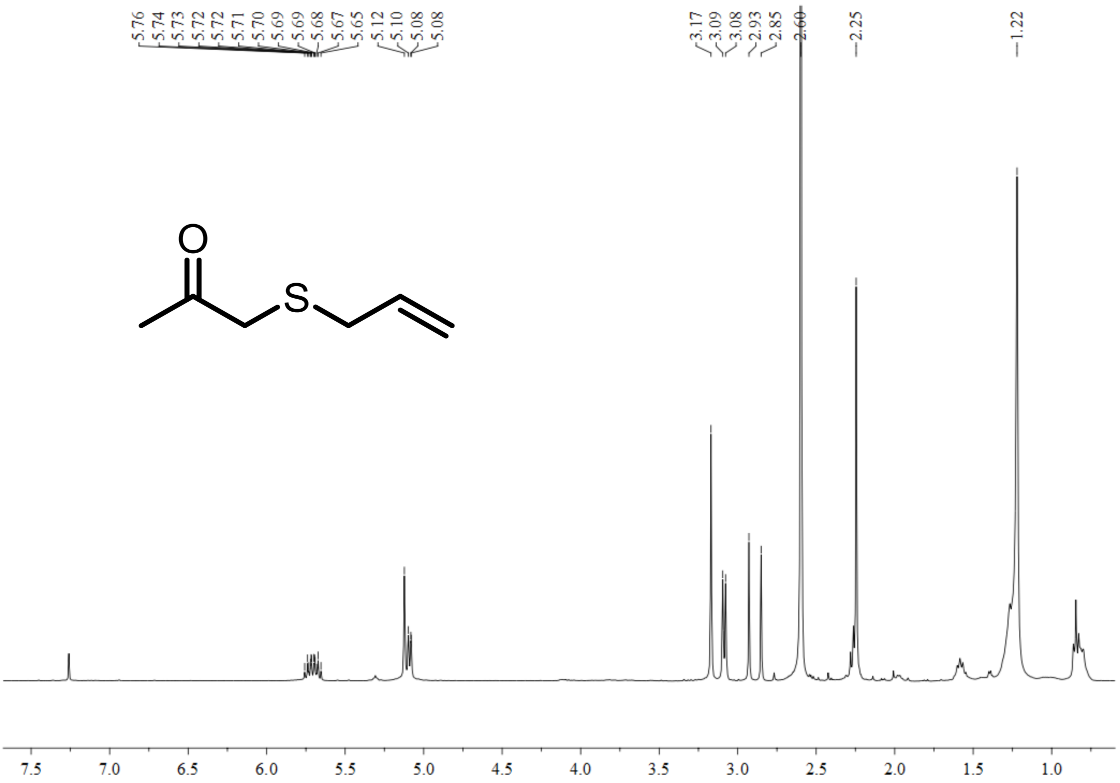

^{13}C NMR 1-(allylthio)propan-2-one (**2v**)

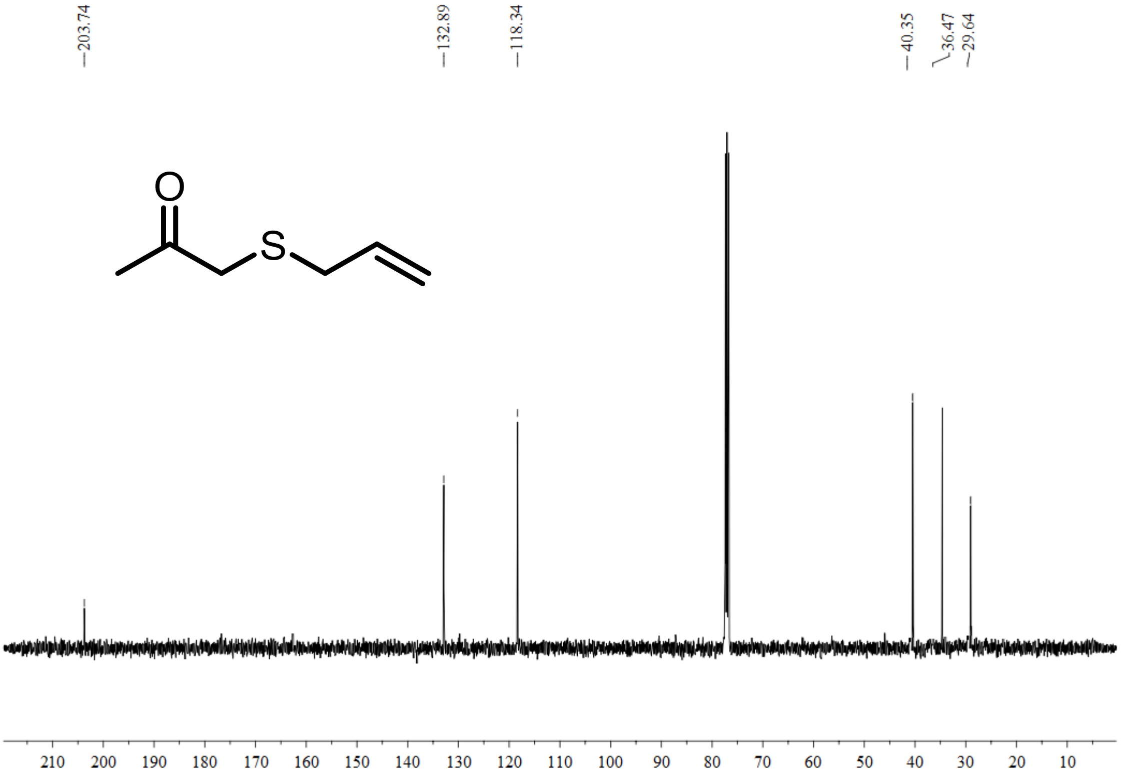

^{1}H NMR (Z)-1-(but-3-en-1-ylthio)prop-1-en-2-yl acetate (**1w**)

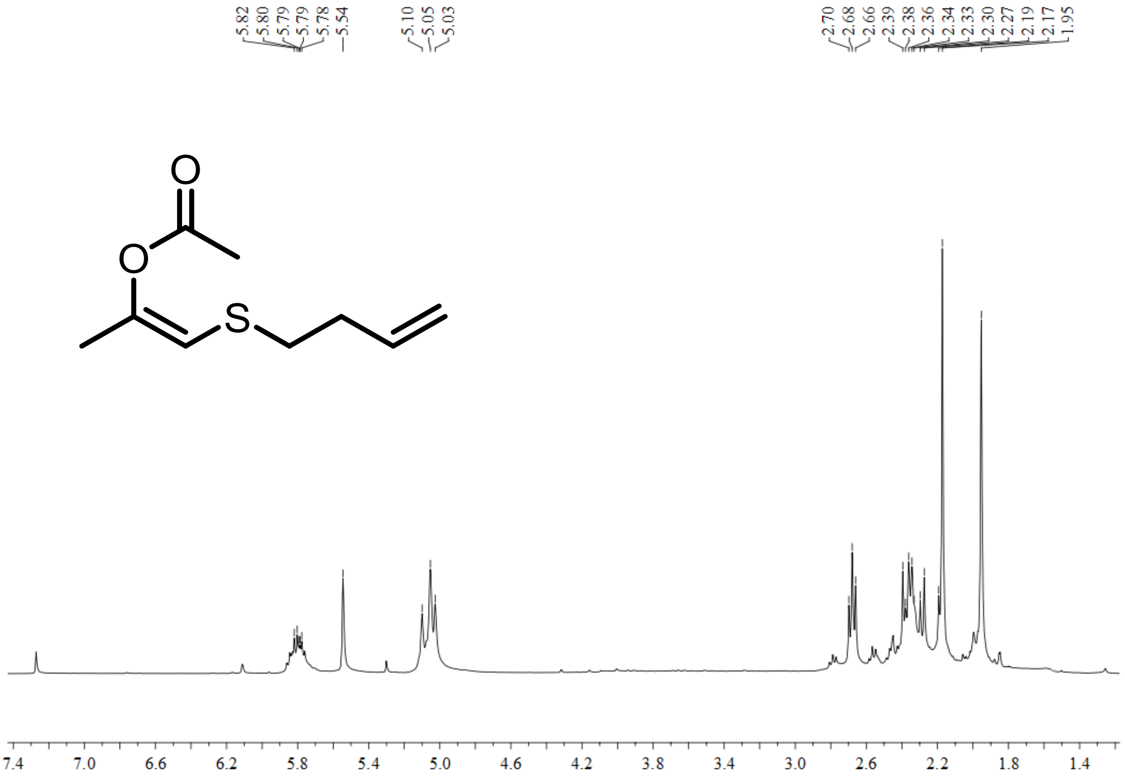

^{13}C NMR (Z)-1-(but-3-en-1-ylthio)prop-1-en-2-yl acetate (**1w**)

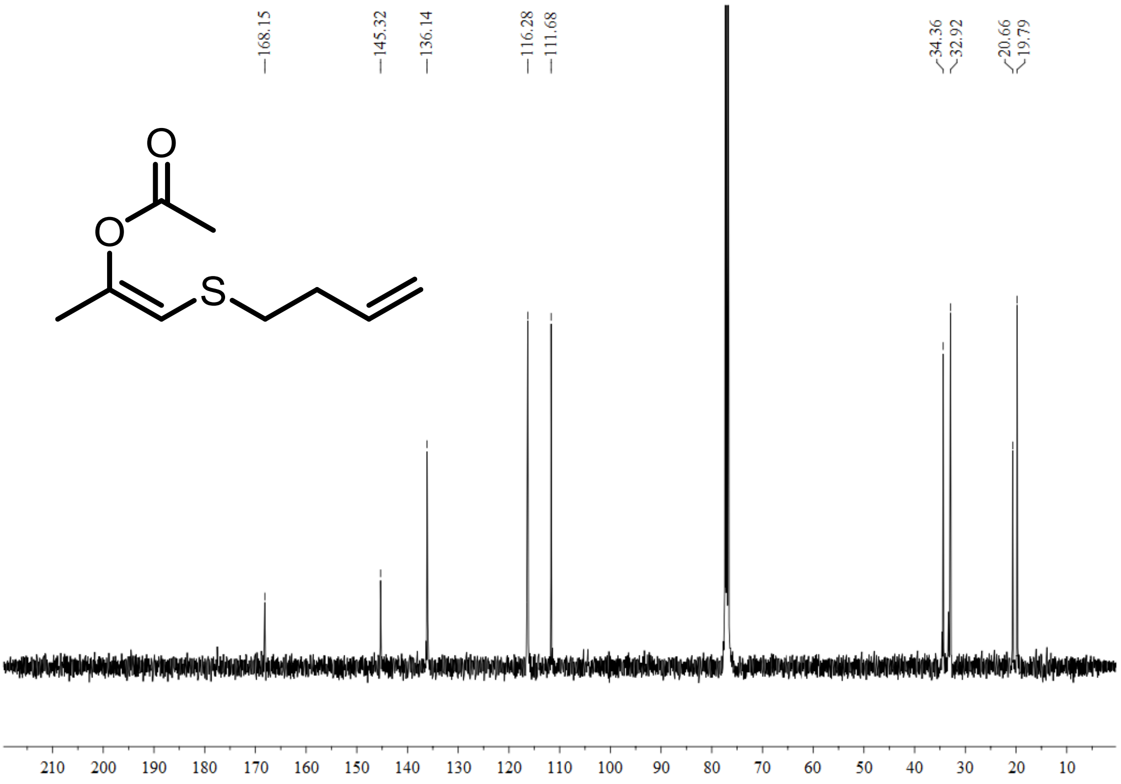

^{1}H NMR 1-(but-3-en-1-ylthio)propan-2-one (**2w**)

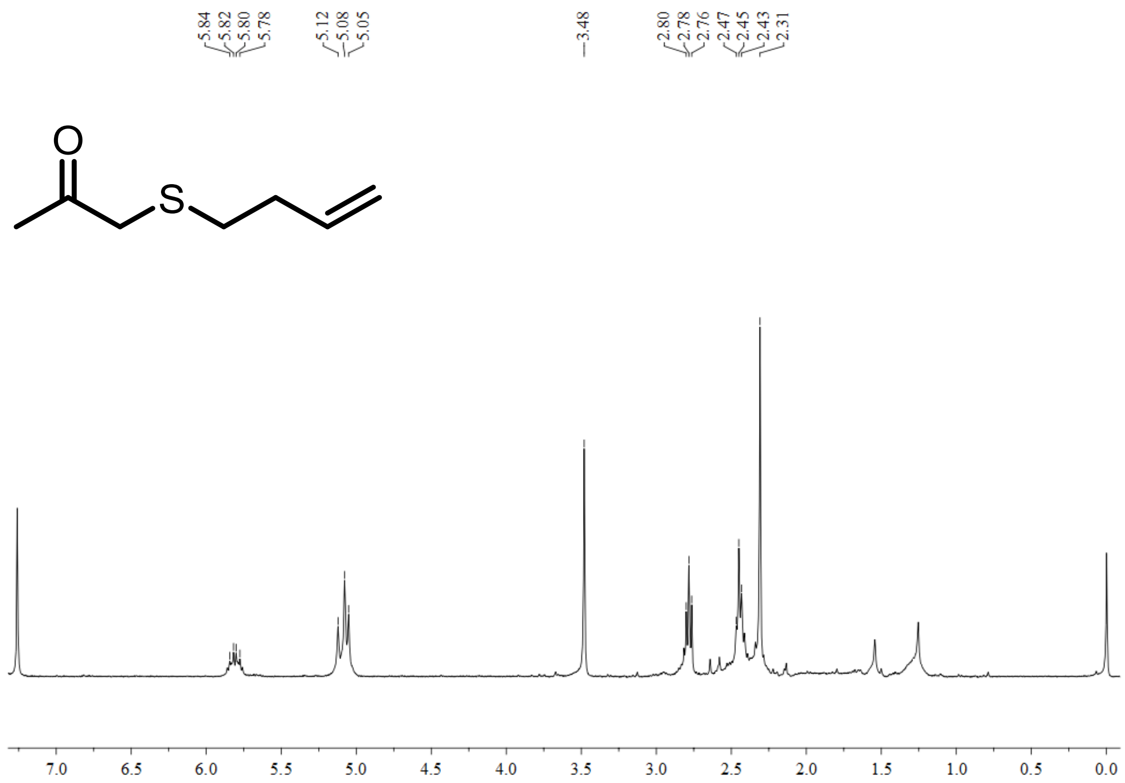

^{13}C NMR 1-(but-3-en-1-ylthio)propan-2-one (**2w**)

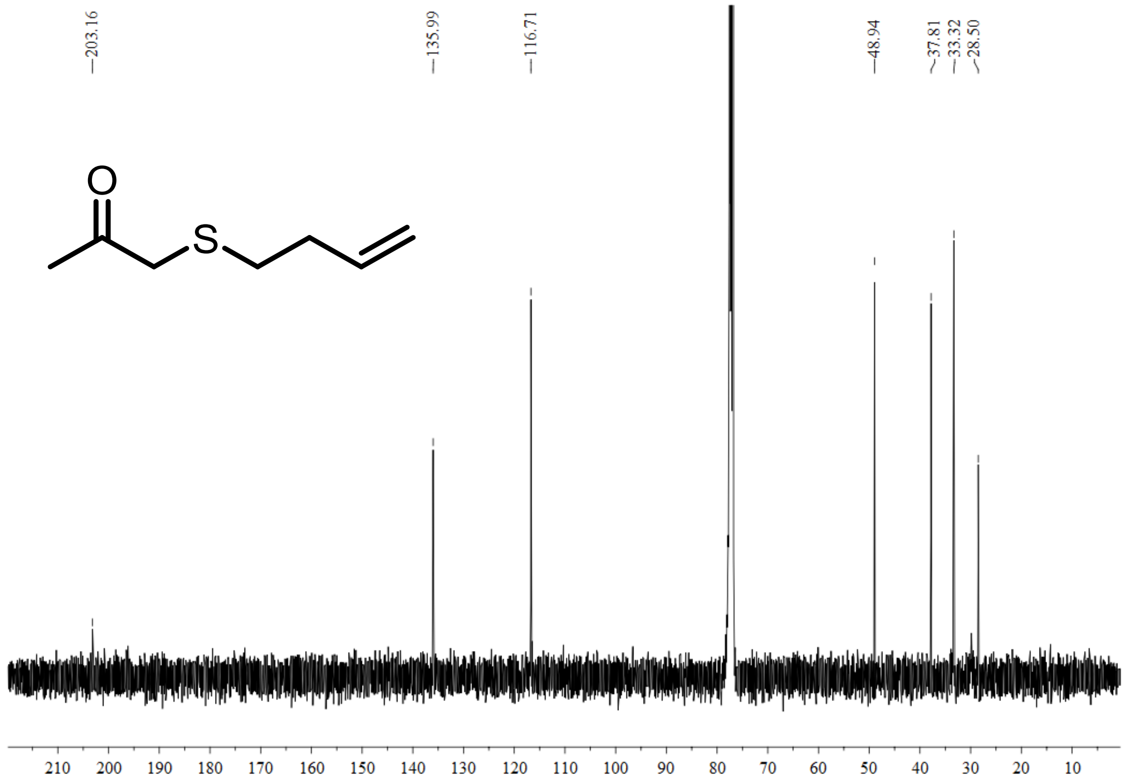

^{1}H NMR (Z)-1-(benzylthio)prop-1-en-2-yl acetate (**1x**)

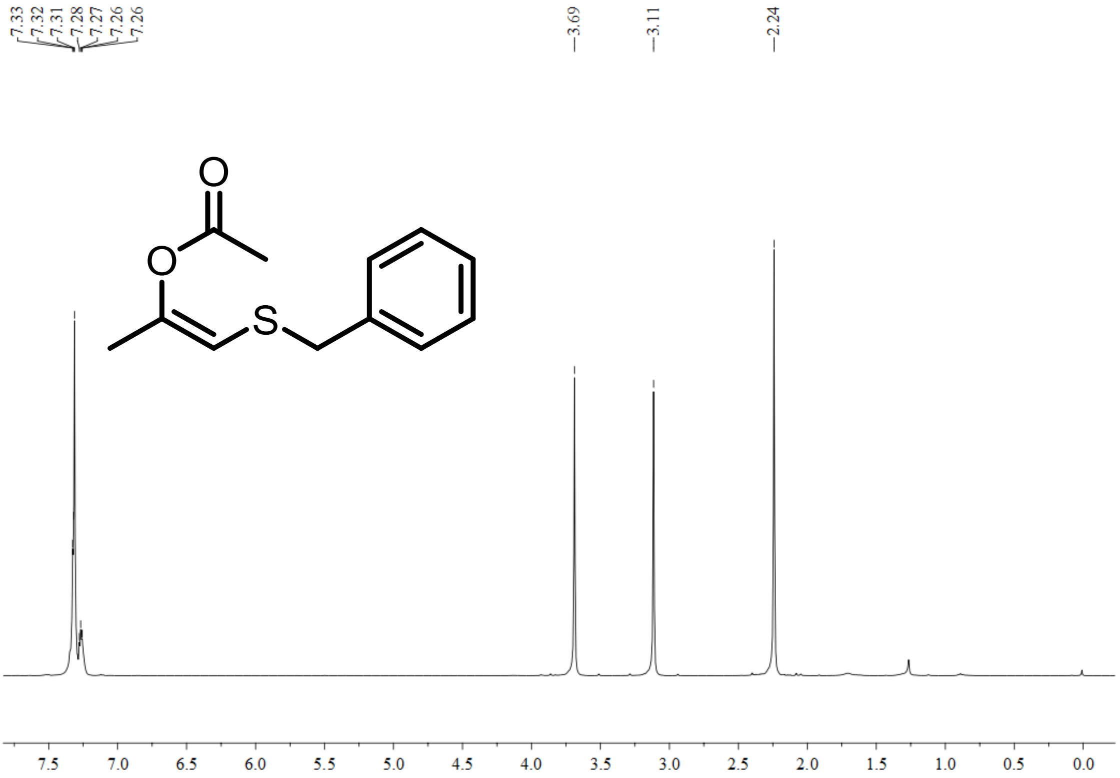

^{13}C NMR (Z)-1-(benzylthio)prop-1-en-2-yl acetate (**1x**)

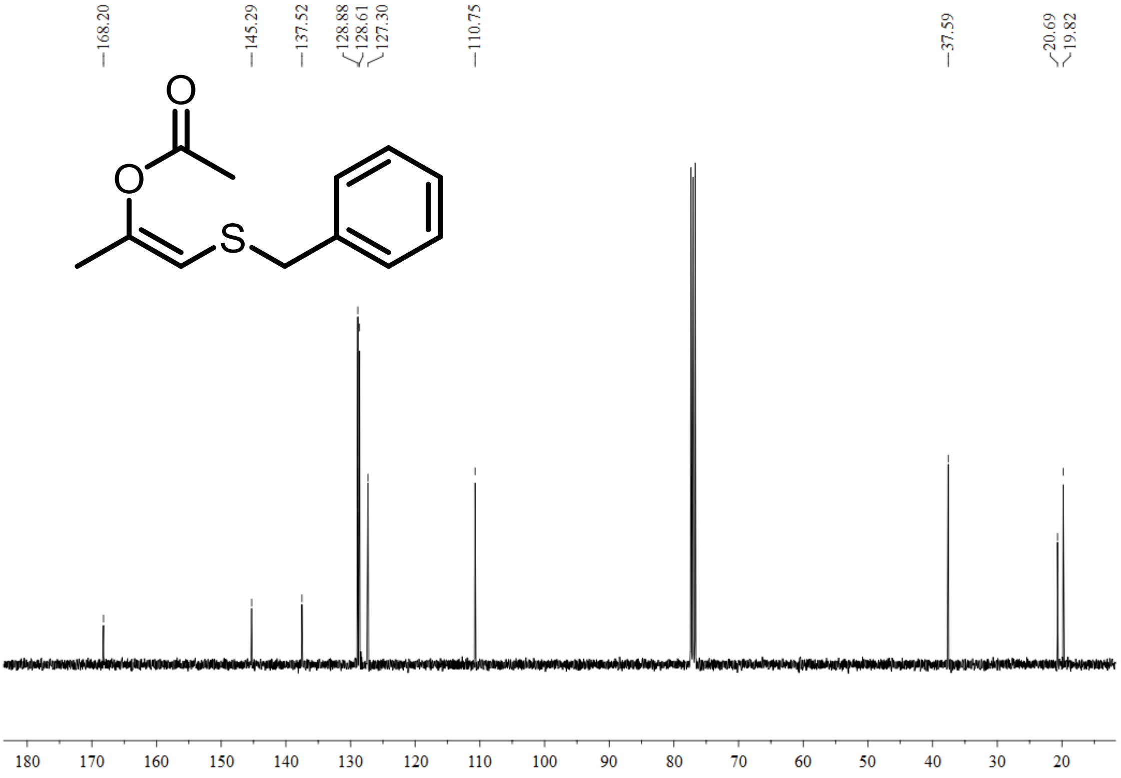

^{1}H NMR 1-(benzylthio)propan-2-one (**2x**)

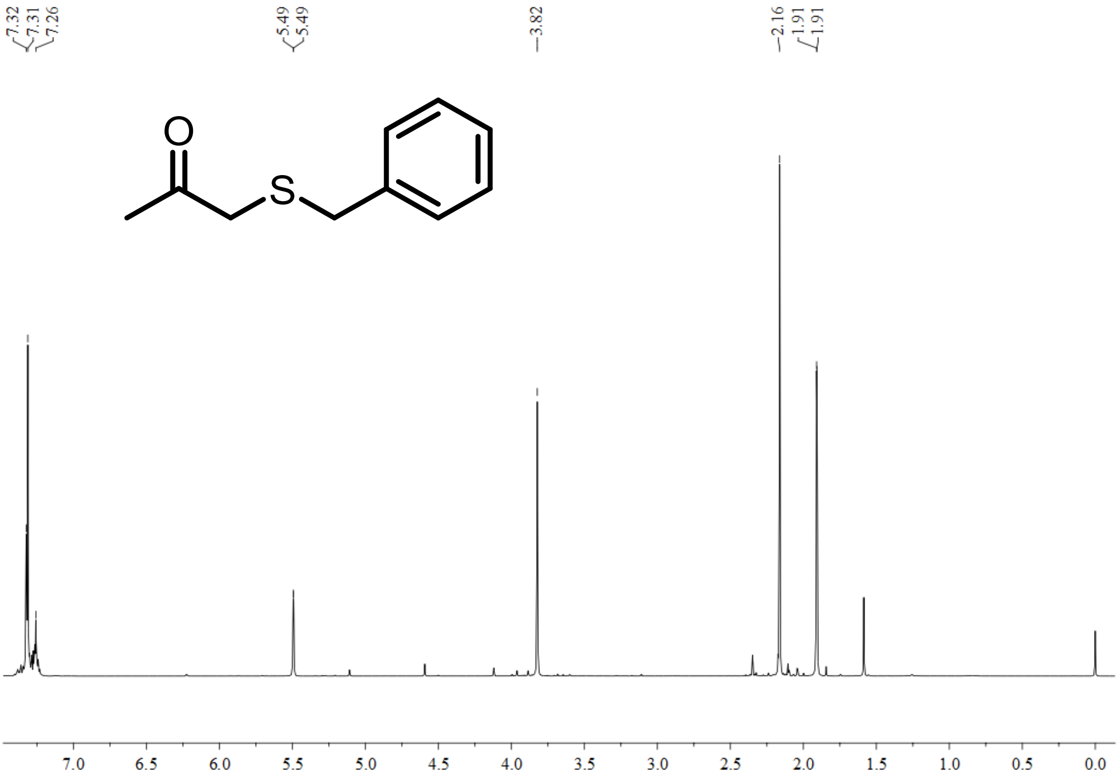

^{13}C NMR 1-(benzylthio)propan-2-one (**2x**)

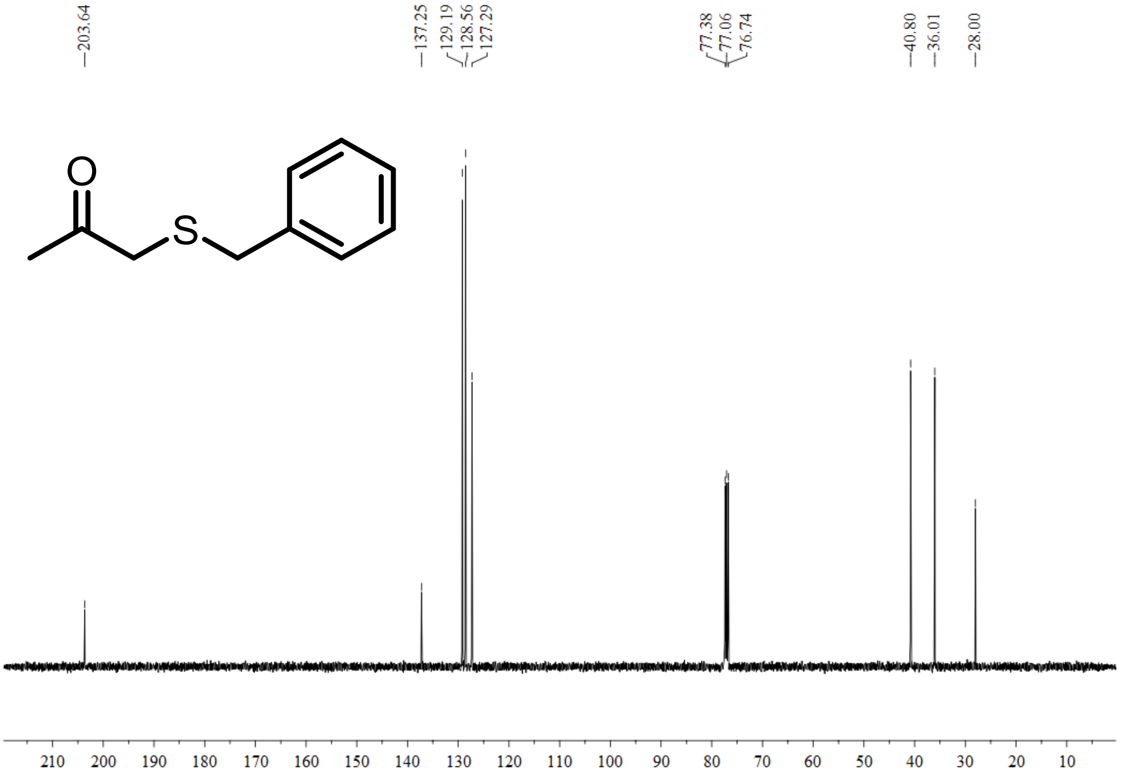

^{1}H NMR 2-(methylsulfinyl)-1-phenylethan-1-one (**3**)

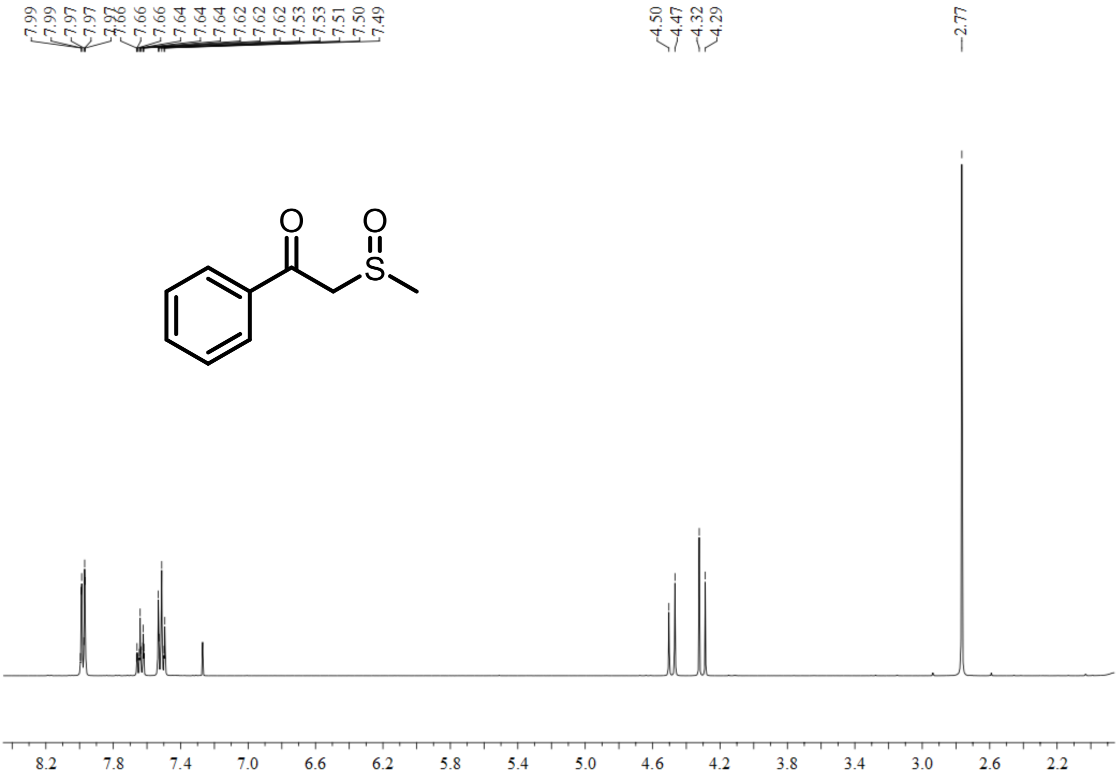

^{13}C NMR 2-(methylsulfinyl)-1-phenylethan-1-one (**4**)

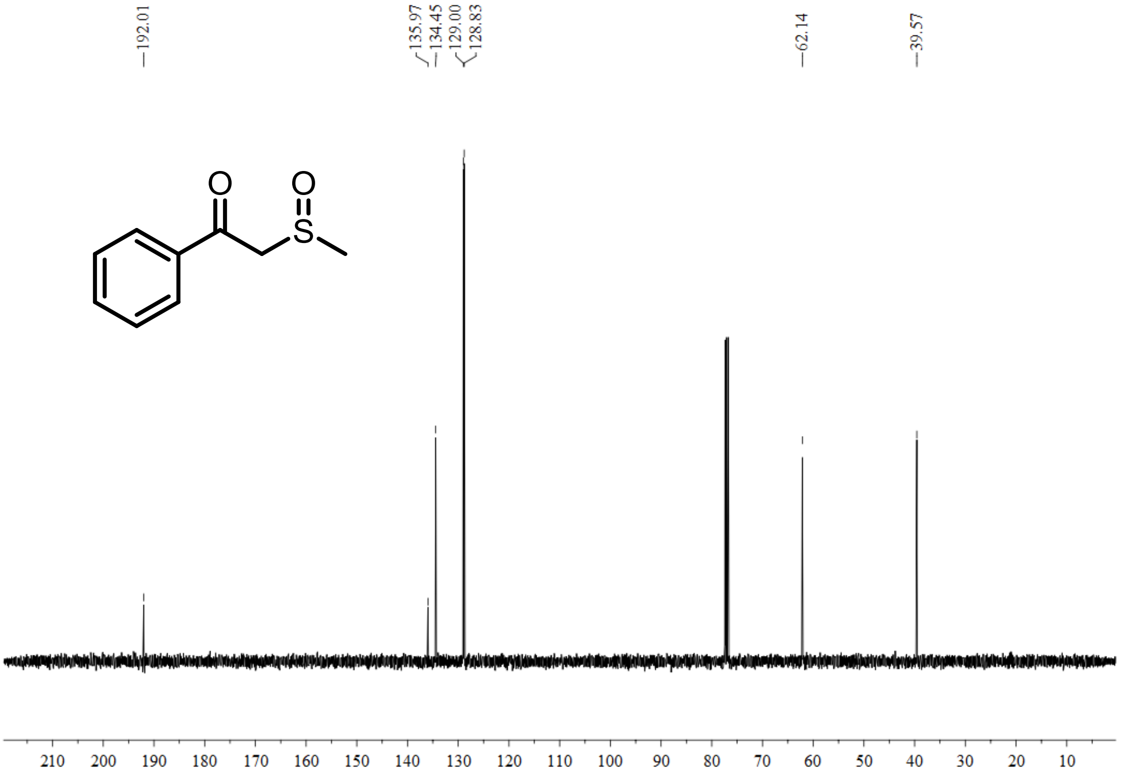

^{1}H NMR 2-(methylsulfonyl)-1-phenylethan-1-one (**4**)

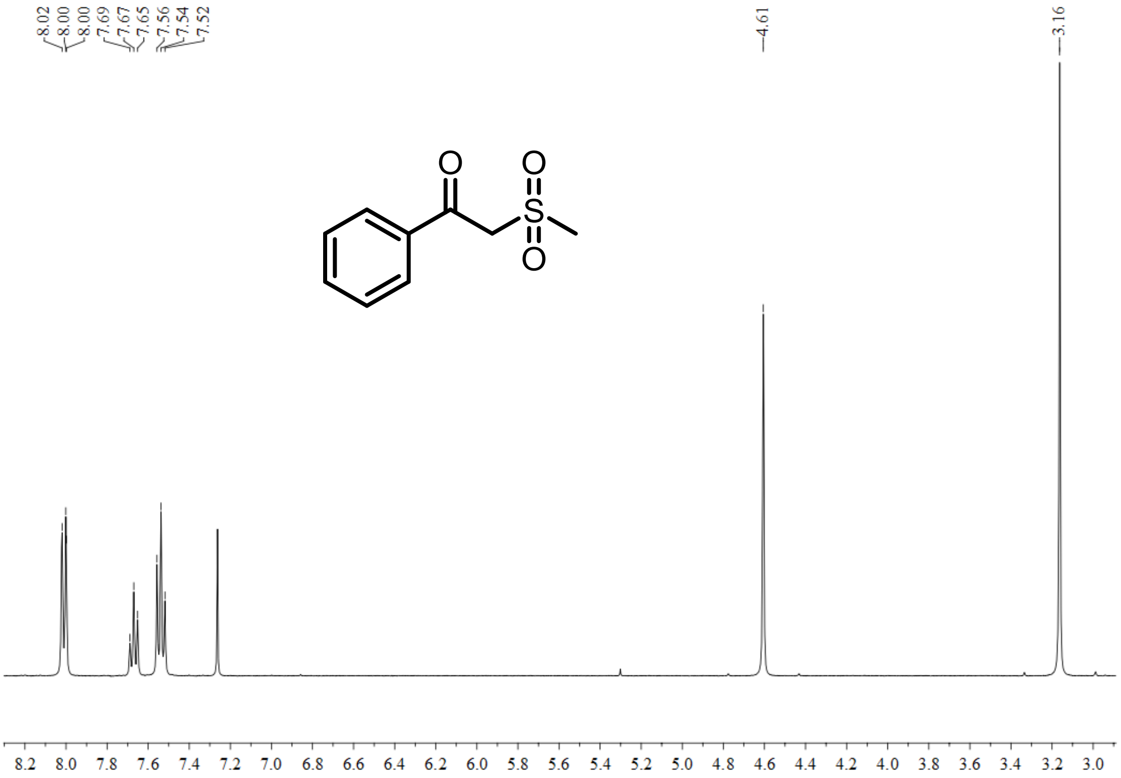

^{13}C NMR 2-(methylsulfonyl)-1-phenylethan-1-one (**4**)

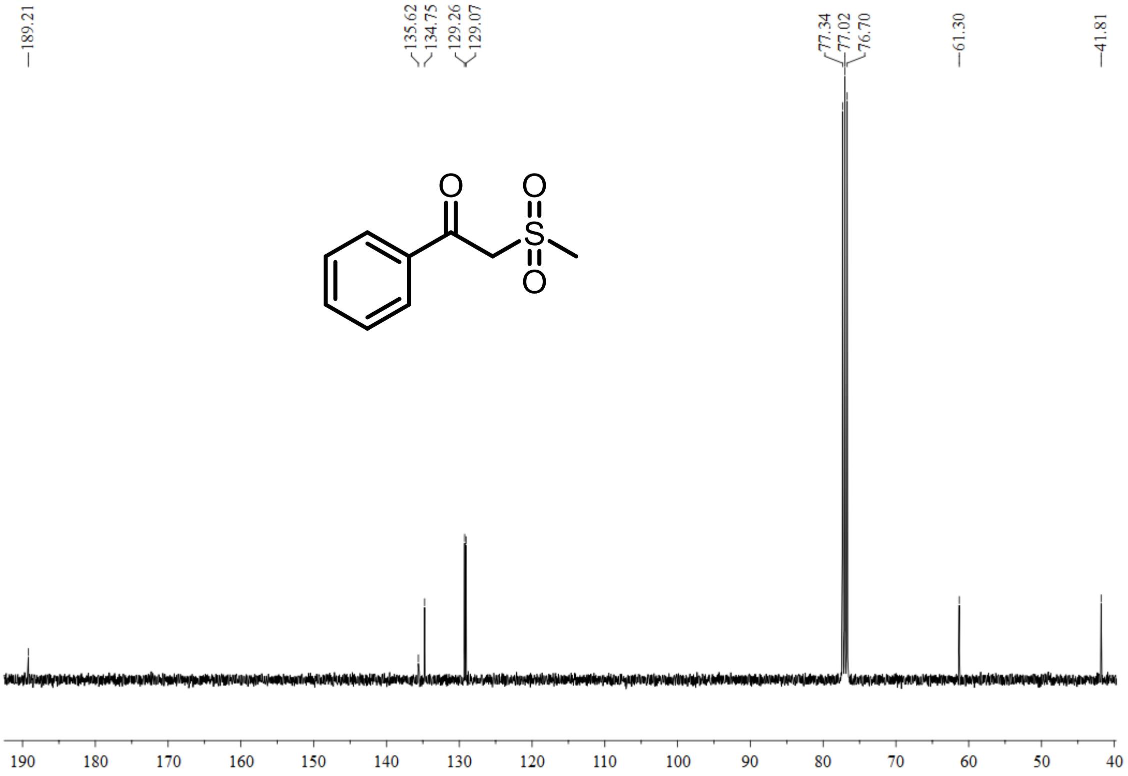

Supplement: File 1 — General procedures and NMR spectra. [file Beilstein_J_Org_Chem-15-378-s001.pdf]
